# An empirical comparison of deep CNN architectures for heart sound classification from phonocardiogram signals

**DOI:** 10.3389/fphys.2026.1927399

**Published:** 2026-08-26

**Authors:** Arar Al Tawil, Bodor Bin Sheeha, Aseel Aburub

**Affiliations:** 1 Department of Computer Science, Faculty of Information Technology, Applied Science Private University, Amman, Jordan; 2 Department of Rehabilitation Sciences, College of Health and Rehabilitation Sciences, Princess Nourah Bint Abdulrahman University, Riyadh, Saudi Arabia; 3 Department of Physiotherapy, Faculty of Allied Medical Sciences, Applied Science Private University, Amman, Jordan

**Keywords:** CNN, deep learning, heart sound, log-Mel spectrogram, phonocardiogram, PhysioNet 2016, transfer learning

## Abstract

Heart disease continues to be the world’s number one killer, making low-cost, early screening a priority. A phonocardiogram (PCG), a simple recording of sounds made by the heart, provides valuable clues to conditions such as murmurs and valve defects and can be recorded cheaply using a digital stethoscope. With less accessibility to specialists, the automatic analysis of heart sounds is preferred for screening and tele-health environments. We compare five deep convolutional neural networks in this work, namely LeNet5, AlexNet, VGG16, ResNet-50 and a hybrid LSTM-CNN, on the task of separating normal from abnormal heart sounds. Before entering the network, each recording is converted to a log-Mel spectrogram, and each model is trained at five different learning rates (1e-2, 1e-3, 5e-4, 1e-4 and 5e-5). The PhysioNet/CinC Challenge 2016 database is common to all experiments and, after duplicate removal, provides 3, 240 recordings. A leakage-free protocol is enforced throughout: exact duplicates are detected by audio content hashing and the split is patient-independent, which on this dataset is sufficient to prevent several networks from reaching a misleading 100% accuracy. We report accuracy, AUC, sensitivity, specificity, F1 score and the Matthews correlation coefficient. Under five-fold cross-validation VGG16 attains the highest mean accuracy (91.94% ± 1.59, AUC 0.968, MCC 0.745), marginally ahead of ResNet-50 (90.93% ± 1.09); McNemar’s test confirms that the difference between the leading architectures is not statistically significant (p = 0.31), so the two are reported as jointly strongest. An ablation study shows that spectrogram augmentation, rather than ImageNet pre-training, is the decisive design choice, and every model fails at a learning rate of 1e-2. External validation on the CirCor DigiScope 2022 database shows that zero-shot cross-dataset transfer performs at chance level, while the retrained pipeline exceeds the majority baseline, underlining how strongly single-dataset results can overstate real-world generalisation.

## 1 Introduction

Heart disease holds the number-one cause of death globally. According to World Health Organization statistics, around 19.8 million people died of cardiovascular disease in 2022. Cardiovascular disease accounted for about 32 percent of all death cases in this year. The brunt is born by low- and middle-income countries where more than three-quarters of these deaths occur and where specialist cardiologists and imaging equipment are typically unavailable (World Health Organization, 2023). In such places, cheap and portable screening tools are essential for the early detection of at-risk patients, not a luxury. The oldest way of checking the heart health is listening to the heart with a stethoscope, a procedure known as auscultation. It is, after all, the first test done by a doctor which costs next to nothing or not much more and requires no special equipment (Liu et al., 2016). The catch is that making this judgment by ear is very subjective, and the end result hinges on the experience of the listener. It therefore does not scale to exhaustive mass screening. By capturing the sounds as a phonocardiogram (PCG), part of that issue is taken away, since the signal can be archived, shared and examined objectively (Liu et al., 2016). A normal heart sound consists of the S1 and S2 sounds. Valve defects and septal defects will add their own characteristic heart murmurs. In addition extra sounds due to turbulence can be identified. Telehealth systems now use automatic machine-learning diagnosis capable of running remotely and at scale. With wearables, digitals stethoscopes and even smartphones capable of recording PCGs, large collections of these has appeared (depending on manufacturer) (Chen et al., 2021). The first systems largely used hand-crafted descriptors (time-domain statistics, spectral features, MFCCs) with classical classifiers, such as support vector machines (SVMs) (Davis and Mermelstein, 1980). These systems tend to rely on careful feature engineering, and rarely transfer well to the messy range of conditions found in real recordings. The introduction of deep learning revolutionized the situation by allowing models to autonomously discern valuable traits (Chen et al., 2021). Convolutional Neural Networks (CNNs) lend themselves naturally to the problem: once a one-dimensional sound is transformed into a two-dimensional time-frequency image or spectrogram, a CNN can read it much as it reads an ordinary photograph (LeCun et al., 1998). The depths of architectures have gradually increased over time, from the initial LeNet5 (LeCun et al., 1998) to AlexNet (Krizhevsky et al., 2012), to the very deep VGG networks (Simonyan and Zisserman, 2015) and the more residual ResNet family (He et al., 2016); hybrid designs that bolt a Long Short-Term Memory block on to the convolutional layers as well to add a sense of time (Hochreiter and Schmidhuber, 1997). Transfer learning has taken results further still because networks trained on the huge ImageNet collection often work well even when the new dataset is tiny (Deng et al., 2009). A significant amount of this research has focused on heart sounds, most of which is based on the PhysioNet/Computing in Cardiology Challenge 2016 database (Clifford et al., 2016; Liu et al., 2016). Potes and his coworkers used feature-based classifiers combined with a one-dimensional CNN (Potes et al., 2016), Nilanon et al. used a two-dimensional CNN net on spectral features (Nilanon et al., 2016), while more recent works added attention modules and richer time-frequency inputs to even further improve accuracy (Li et al., 2022; Huai et al., 2025). Nonetheless, there are two challenges that arise. Most studies evaluate one architecture in one setting only. Therefore, it is difficult to say how the main CNN families really compare when everything else is held equal. The learning rate is seldom a subject of inquiry by itself, and the peril of data leakage from duplicated recordings is almost never discussed: this can make results seem far better than they really are. In this work, we take up both problems in a controlled comparison run under a strict, repeatable protocol, building on our earlier work on audio classification with deep CNNs (Almazaydeh et al., 2022). Our contributions are fourfold: we conduct a comparison of five representative CNN architectures (LeNet5, AlexNet, VGG16, ResNet-50 and a hybrid LSTM-CNN) on normal-versus-abnormal heart-sound classification using PhysioNet 2016; we study how the learning rate affects each of them across five values; we introduce a leakage-free protocol whereby the contents are hashed across all recordings so that duplicate recordings are eliminated prior to splitting and patients never cross between sets; and everything is evaluated based on six metrics and not accuracy alone.

The paper is arranged as follows. Sections 2, 3, 4 and 5 are literature review, data and methods, results and discussion, and conclusion, respectively.

## 2 Related works

Research on automatic heart-sound analysis has been carried out for several decades, but the field gained real momentum once large shared datasets became available. The release of the PhysioNet/Computing in Cardiology (CinC) Challenge 2016 was a watershed moment: for the first time researchers had a single large benchmark of normal and abnormal phonocardiograms drawn from many sources, with an agreed scoring rule, which made results from different groups directly comparable (Clifford et al., 2016; Liu et al., 2016). The database has been built to be deliberately heterogeneous, with recordings captured with different stethoscopes and recorded in quiet clinics through to noisy field conditions, so generalisation is a real challenge rather than an afterthought. Before deep learning came into being, most systems had a two-stage recipe. Hand-crafted descriptors were first extracted from the signal, usually a mixture of time-domain statistics, spectral measures and Mel-Frequency Cepstral Coefficients (MFCCs) (Davis and Mermelstein, 1980). These features were then fed to a conventional classifier, such as a support vector machine, a k-nearest-neighbour rule, a random forest or a boosted ensemble. Although such pipelines performed well on clean recordings, they relied heavily on expert feature design and degraded when the audio was noisy or came from an unseen device. The 2016 challenge accelerated the shift toward learned features. The winning entry combined a bank of handcrafted features with a one-dimensional convolutional network inside an AdaBoost ensemble. It was developed by Potes et al. and achieved an overall challenge score of around 0.86 (sensitivity 0.94, specificity 0.78) (Potes et al., 2016). Other strong contenders reformulated the task as an image classification task: Rubin et al. segmented each recording, turned it into an MFCC heat map and classified it with a deep CNN (Rubin et al., 2016), while Nilanon et al. applied a 2D CNN directly to spectral representations of the beats (Nilanon et al., 2016). Soon after, Maknickas and Maknickas demonstrated that a deep CNN adeptly fed with Mel-frequency spectral coefficients could reach competitive accuracy on unsegmented recordings, which in turn made unnecessary an explicit segmentation stage (Maknickas and Maknickas, 2017). These systems developed using early learned features were already better than most of the purely hand-engineered pipelines. They set the blueprint that most of the later work still undoubtedly follow, including our own: turn the phonocardiogram into a spectrogram-like image and let a convolutional network learn the differentiating patterns. Aside from the classifier selection, the studies differed in signal preparation. Some segmented each recording into individual S1-S2 cycles before running the analysis, while others worked on whole unsegmented short clips. Besides, the input signal also varied: it included cepstral coefficients, Mel spectrograms and wavelet scalograms. This variation, albeit yielding, is exactly what renders a head-to-head comparison so challenging, because of how different two methodologies could be, as much in their front-end as in their network. After the challenge, the focus moved away from straightforward CNN applications to enhancements in accuracy, lightness or robustness. In terms of efficiency, Xiao et al. devised a one-dimensional CNN with a surprisingly low number of parameters. Its strong performance shows that efficiency does not always require a heavy computational cost, which is desirable for wearable or embedded use (Xiao et al., 2020). Others pursued unrefined precision. Deperlioglu fed signal instant-energy features to a stacked autoencoder and produced figures above 99 percent (Deperlioglu, 2021), Li et al. paired an improved Mel-frequency spectral representation with deep residual learning to reach roughly 94 percent (Li et al., 2022) and Huai et al. added a Convolutional Block Attention Module into a CNN so that the network could weight the most informative time-frequency regions, lifting accuracy as high as 98.66 percent (Huai et al., 2025). Most recently studies confirm that convolutional and hybrid convolutional-recurrent models take over much of the literature, and that spectrogram-style inputs are by far most common representation (Chen et al., 2021). Despite all the above advances, three weaknesses re occur. A large number of papers are evaluating a single architecture tuned to a single configuration, making it hard whether a gain reported is from the network of the paper or the preprocessing, feature extraction or training schedule; controlled comparisons of several architectures under identical conditions remain rare. Beyond the choice of a model, the learning rate, arguably the most important training hyper-parameter, is seldom reported in detail and almost never studied as a variable, although it strongly affects stability and final accuracy. Most worryingly for reproducibility, many papers are vague about a key step they took to stop information leaking from training to test data. The PhysioNet 2016 collection contains near-duplicate and duplicated recordings, and a single patient may contribute several files. If the data are split at the clip level rather than the patient level, or if duplicates are left in place, a model can score almost perfectly simply by recognising material it has already seen. Therefore, the very high accuracies that are sometimes reported on this dataset should be viewed with caution, as they are not necessarily obtained under comparable or leakage-controlled conditions. The focus of this research paper is specifically on these three shortcomings. To begin with, this paper compares five established CNN architectures, all within one fixed pipeline. Next, it treats the learning rate as an explicit experimental factor. Finally, it enforces a strict, duplicate-free and patient-independent evaluation. Thus, the numbers reported in this paper are comparable and reproducible. The present work can be placed alongside prior work as summarised in **Table 1**. The literature has continued to move quickly. Recent work has concentrated on attention mechanisms, hybrid transformer designs and lightweight ensembles: Huai et al. (2025) integrated a convolutional block attention module and reported accuracy approaching 98.7%, Al-Tam et al. (2024) combined a transformer encoder with a residual convolutional branch, Rong et al. (2024) proposed a light-weight attention-based ensemble aimed at deployment, and Ay and Gunes (2025) reported further gains from a redesigned deep pipeline in 2025. A 2024 review by Chen et al. (2024) confirms that convolutional and hybrid convolutional-recurrent models continue to dominate the field and that spectrogram-style inputs remain the most common representation. What these studies rarely make explicit, however, is the partitioning protocol under which their figures were obtained, which is precisely the gap addressed here.

**Table 1:** Summary of representative prior studies on heart-sound classification and the position of this work.

Study	Year	Input features	Model	Reported accuracy
Potes et al. (2016)	2016	MFCC + time/frequency features	1D-CNN + AdaBoost ensemble	0.86 (challenge score)
Rubin et al. (2016)	2016	MFCC heat maps	Deep 2D-CNN	Challenge entry
Nilanon et al. (2016)	2016	Spectral features	2D-CNN	Challenge entry
Maknickas and Maknickas (2017)	2017	MFSC (unsegmented)	2D-CNN	~0.84
Xiao et al. (2020)	2020	Raw PCG	Lightweight 1D-CNN	~0.93
Deperlioglu (2021)	2021	Signal instant energy	Stacked autoencoder	~0.996
Li et al. (2022)	2022	Improved MFSC	Deep residual CNN	0.944
Huai et al. (2025)	2025	Log-Mel spectrogram	CNN + CBAM	0.987
This work	2026	Log-Mel spectrogram	Five CNNs (best ResNet-50)	0.934 (leakage-free)

## 3 Materials and methods

**Figure 1:**
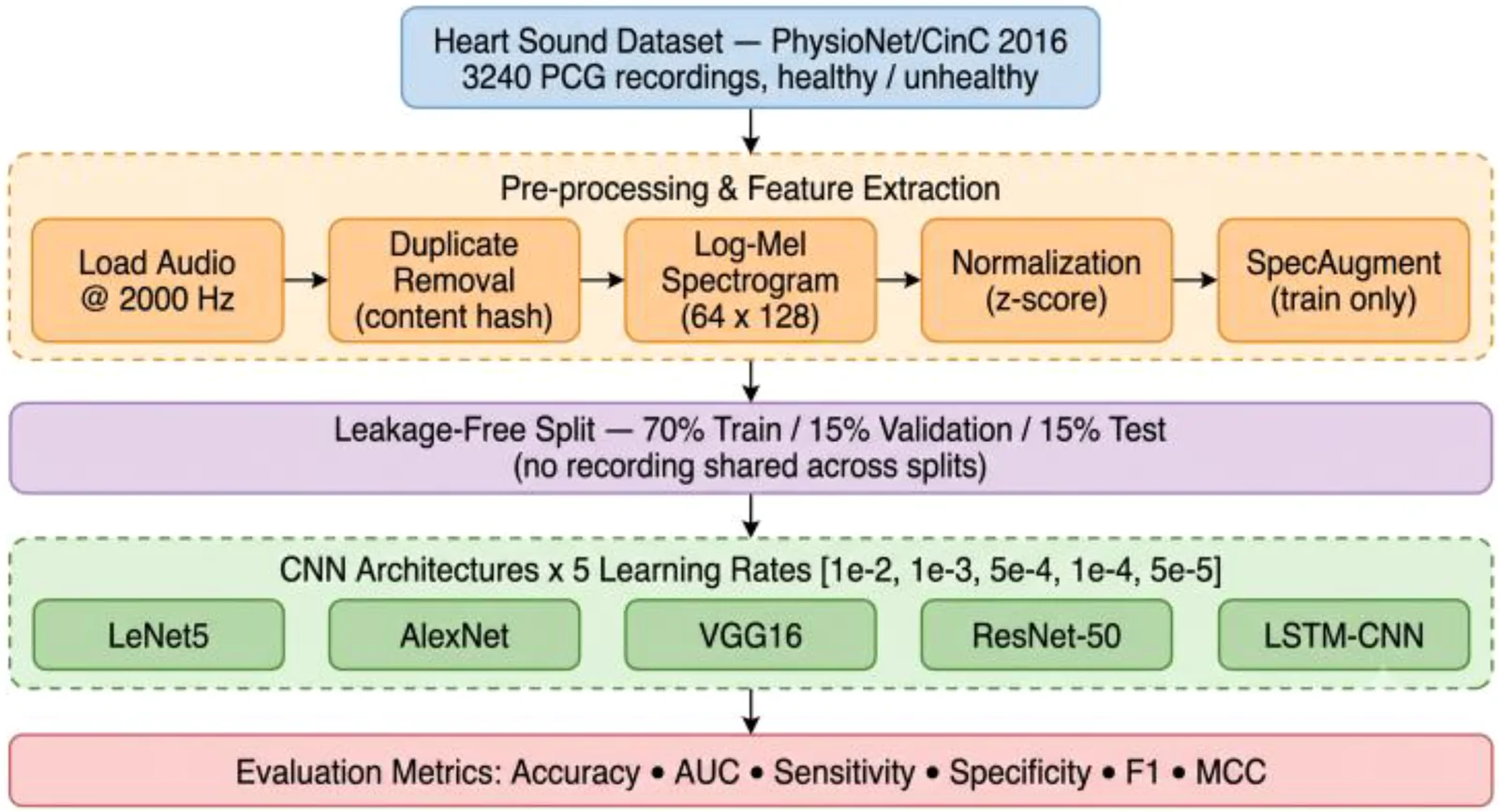
Overall framework of the proposed heart-sound classification study.

This section walks through the methodology shown in **Figure 1**, from preparing the dataset and extracting features, through the leakage-free split, to the CNN models used for training and testing.

### 3.1 Dataset

The PhysioNet/CinC Challenge 2016 heart-sound database is the largest public PCG collection in the world and is used for all of the experiments. Due to the collection of recordings from different independent sources, captured with various digital stethoscopes under clinical and everyday conditions, it embodies a fair amount of real-world variability. Every file is marked as normal (healthy) or abnormal (unhealthy), whereas, the abnormal files come from patients suffering from specific diseases like valve defect or coronary artery disease. We were resampling every recording to 2000 Hz, which threw away no important energy since the useful energy in heart sounds is below 1000 Hz. After filtering out exact duplicates (see Section 3.5), we ended up with 3, 240 recordings – 2, 575 from the normal class and 665 from the abnormal class. The dataset major attributes are given in **Table 2**.

**Table 2:** Composition and properties of the heart-sound dataset.

Property	Value
Source database	PhysioNet/CinC Challenge 2016
Signal type	Phonocardiogram (PCG), WAV
Sampling rate	2, 000 Hz
Original recordings	3, 541
Duplicate recordings removed	301
Unique recordings used	3, 240
Normal (healthy)	2, 575
Abnormal (unhealthy)	665
Train/Validation/Test	2, 268/486/486
Model input (log-Mel spectrogram)	64 x 128

### 3.2 WAV file

**Figure 2:**
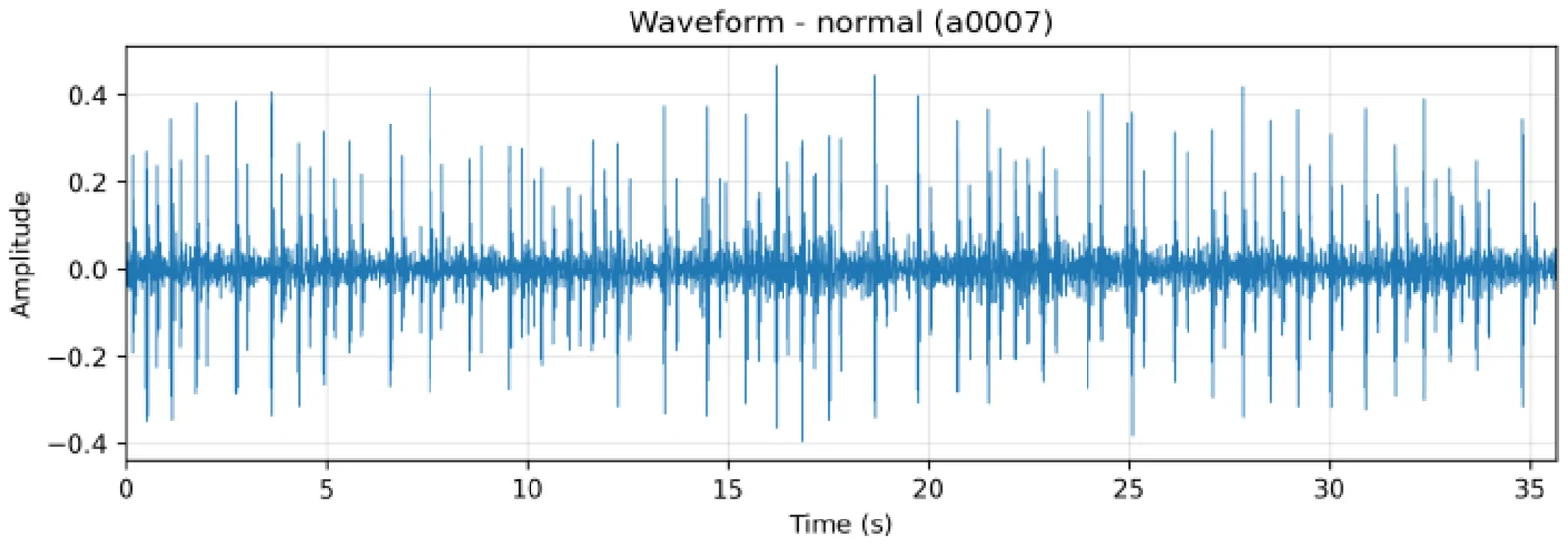
Waveform of a normal heart-sound recording.

**Figure 3:**
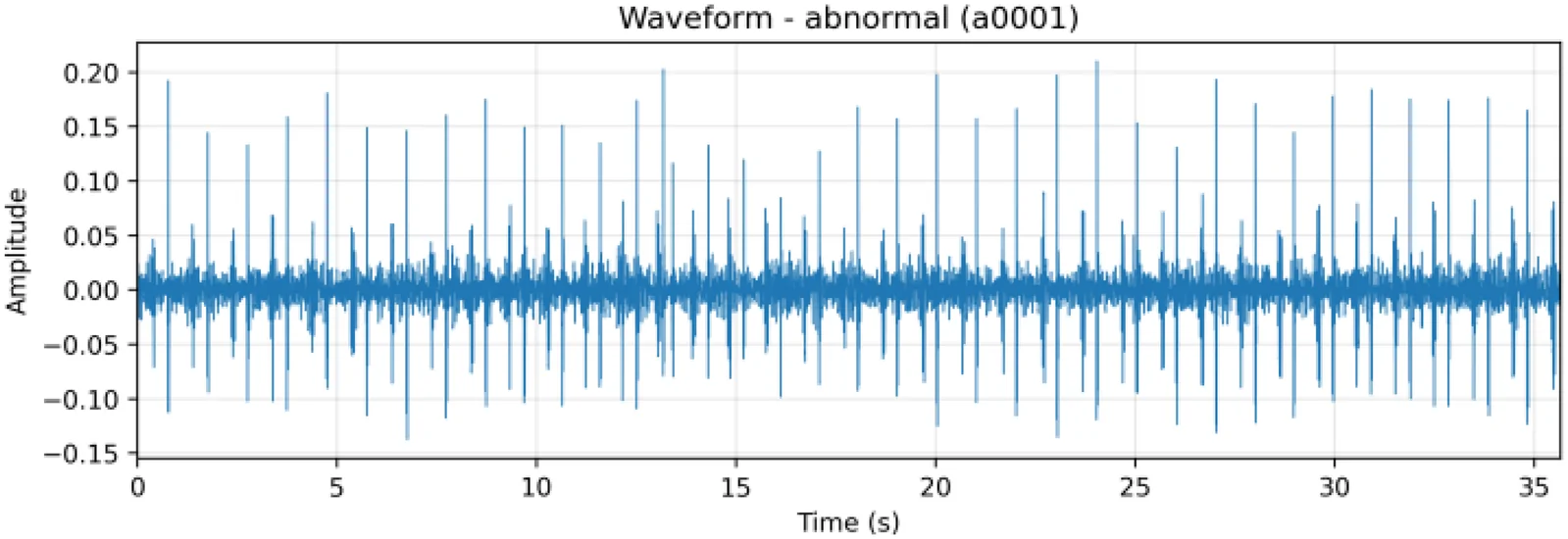
Waveform of an abnormal heart-sound recording.

It all starts with the RAW WAV files, which hold the uncompressed waveform. A graphical representation of cycles of a wave without distortion. As **Figures 2** and **3** show the repeating S1-S2 pattern is easy to recognize, yet a normal and abnormal recording look much alike to the eye. This is precisely the reason we need a time-frequency view and learned features rather than a raw trace.

### 3.3 Short-time fourier transform

**Figure 4:**
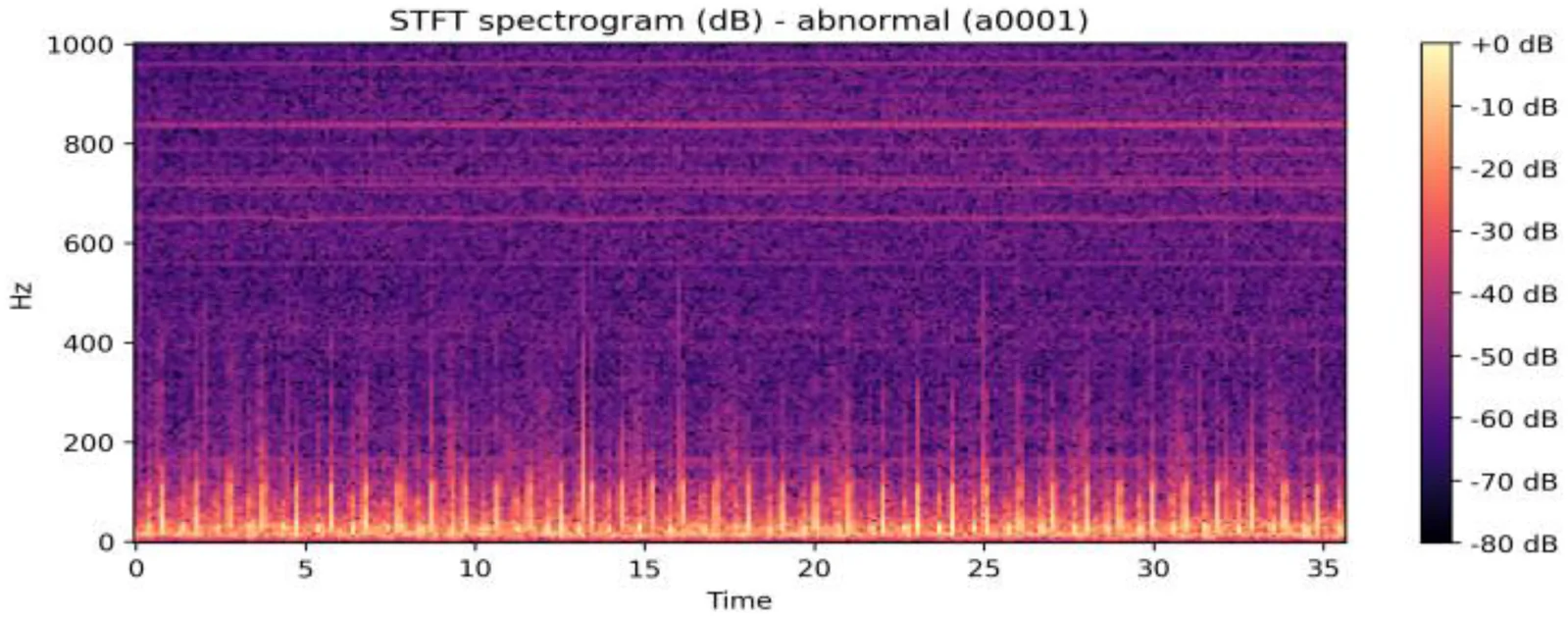
STFT spectrogram (in dB) of an abnormal heart-sound recording.

The Short-Time Fourier Transform (STFT) illustrates how a signal’s spectrum varies over time. The process is as follows: sliding a window across the signal, acquiring the Discrete Fourier Transform of the data within the window, and finally stacking this information together. The resulting data produced from the STFT is frequently depicted in spectrograms. Our sampling rate was 2, 000 Hz, our window (n_fft) 512, and our hop length 256 as shown in **Figure 4**, the periodic beats stand out in low frequencies where most of the energy in a heart sound sits, giving the network much more to play with than the raw waveform.

### 3.4 Log-Mel spectrogram features

**Figure 5:**
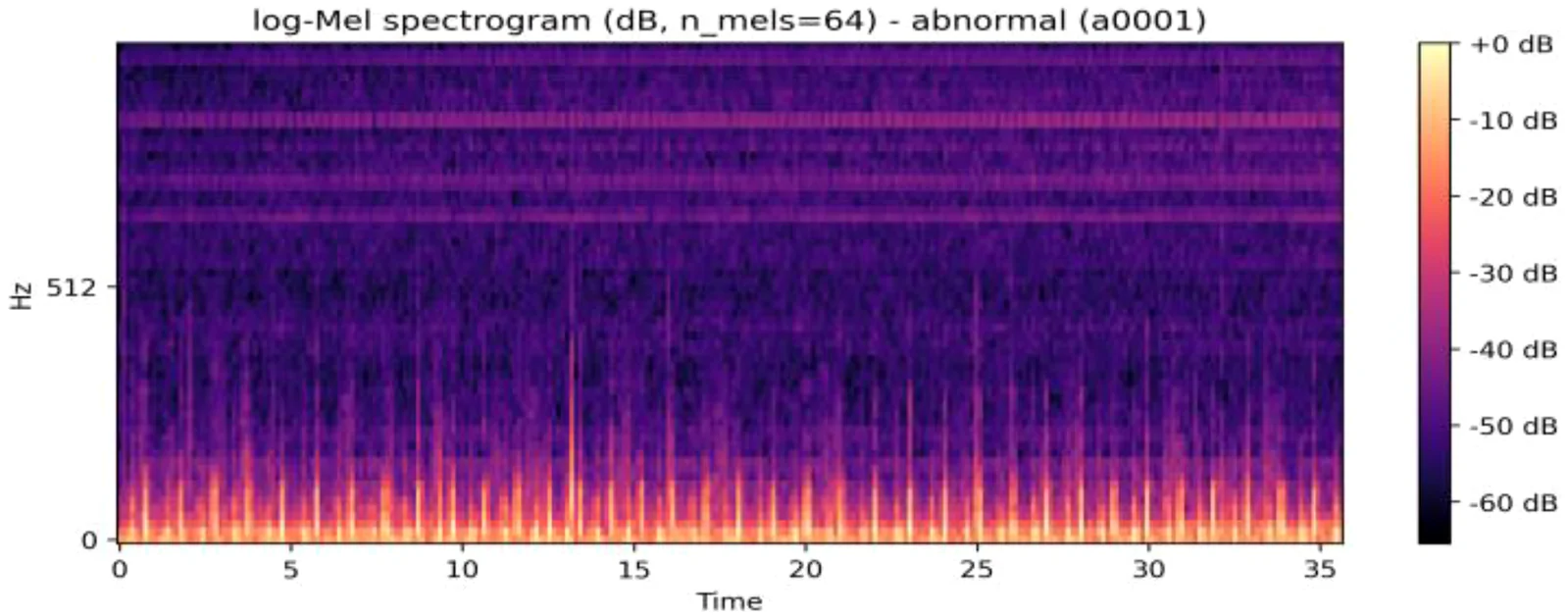
Log-Mel spectrogram (model input) of an abnormal heart-sound recording.

Hearing humans are roughly logarithmic in frequency, so the linear STFT spectrogram is fed into a bank of triangular Mel filters, then converted to decibels. The output will be the log-Mel spectrogram, which is a compact representation of the valuable time-frequency content for ear. Each recording was a log-Mel spectrogram of 64 Mel bands that was either padded or trimmed to 128 frames resulting in a single-channel image of size 64x128. An example is illustrated in **Figure 5**, which shows the data actually received by the networks. Audio processing and feature extraction were performed using the библиотеки librosa (McFee et al., 2015).

### 3.5 Pre-processing and leakage-free protocol

The leakage-free split is the most important part of our pipeline. Audio datasets widely available through the public domain on heart sounds typically include identical recordings in more than one folder. If the data is shuffled too naively, identical clips can end up in both training and test sets. Consequently, the model only memorises them and the scores appear perfect. To avoid this, we hashed the decoded audio of every recording and discarded exact copies this removed 301 files. Afterward, we split the remainder of the recordings into a training (70%), validation (15%) and test (15%) set in a stratified and patient independent manner and checked explicitly that no recording appeared in more than one set. Because the two classes are very unbalanced, during training we augmented the minority class with SpecAugment-style masking of time and frequency (Park et al., 2019), along with a small amount of additive noise, and we weighted the loss by class frequencies. All spectrograms were normalized by the mean and standard deviation of the training set.

### 3.6 Convolutional neural networks

The CNNs are made for grid-like data like pictures, and CNNs treat a spectrum the same way they would a picture. The convolutional layers learn features with trainable filters, and non-linear activations (usually ReLU) increase expressiveness and pooling or sub-sampling layers reduce the spatial size. The five networks are illustrated in **Table 3** and differ in depth. The transfer learning was employed for the two deepest networks VGG16 and ResNet-50. This means that the networks were constructed by initialising from their ImageNet weights. The input 64x128 spectrogram was expanded to three channels as per the requirements of the networks. Subsequent sections will provide details about each model. Sketch the structures from 6 to 10.

**Table 3:** Configuration and parameters of the five CNN architectures (input shape 64 x 128).

Model	Type	Key layers	Pretrained	Parameters
LeNet5	Shallow CNN	2 conv (6, 16 @5x5) + 3 FC	No	~0.82 M
AlexNet	Deep CNN	5 conv + 3 FC	No	~12.76 M
VGG16	Very deep CNN	13 conv (3x3) + GAP	Yes (ImageNet)	~14.72 M
ResNet-50	Residual CNN	49 conv + GAP, skip connections	Yes (ImageNet)	~23.59 M
LSTM-CNN	Hybrid CNN-RNN	2 conv + LSTM(128)	No	~0.61 M

### 3.7 LeNet5

**Figure 6:**
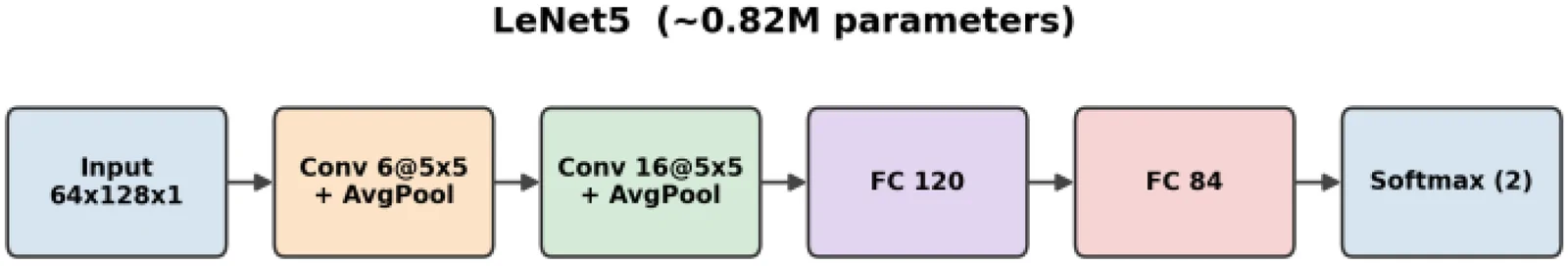
Structure of the LeNet5 architecture.

LeNet5, the early network of LeCun et al. (1998), is the simplest model here. In our setup it uses two convolutional layers of 6 and 16 filters (5x5), each followed by average pooling, then three fully connected layers of 120, 84 and 2 units. On the 64x128 input it holds only about 0.82 million trainable parameters, which makes it a fast, lightweight baseline. **Figure 6** shows its layout.

### 3.8 AlexNet

**Figure 7:**
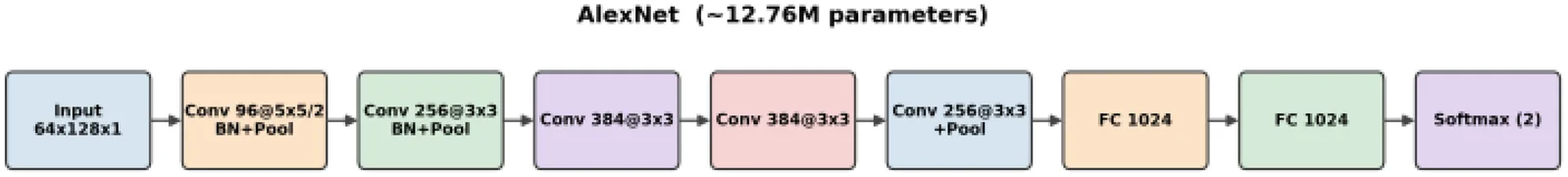
Structure of the AlexNet architecture.

AlexNet became the first winner of the ImageNet competition and inspired large interest into deep CNNs from Krizhevsky et al. (2012). The architecture consists of 5 convolutional layers, each with ReLU activation, batch normalisation, and max pooling. In total, the first layers have (96, 256, 384, 384 and 256 filters). Finally, there are two 1024 fully connected layers with softmax. It roughly has 12.76 million parameters as set up here. In **Figure 7** shown its structure.

### 3.9 VGG16

**Figure 8:**
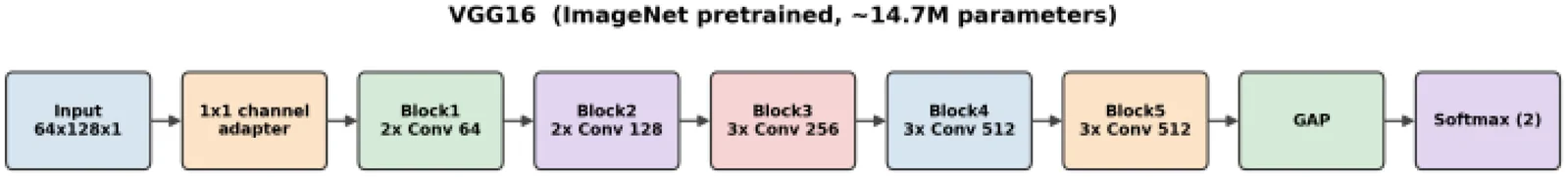
Structure of the VGG16 architecture.

According to Simonyan and Zisserman, VGG16 obtains its great depth by using very small patch sizes (3x3), repeated several times, over 5 blocks and a total of 13 convolutional layers. Thus, the small filter size keeps each layer cheap. Yet the layers are still powerful enough to capture fine details in the image. We began with ImageNet weights and refined on the spectrograms. With 14.72 million parameters in its convolutional base, it is completed by global average pooling and a softmax. The structure is illustrated in **Figure 8**.

### 3.10 ResNet-50

**Figure 9:**
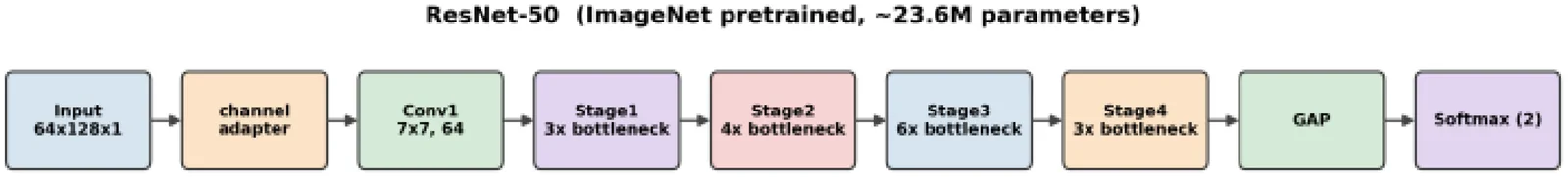
Structure of the ResNet-50 architecture.

He et al. (2016) released ResNet-50, a 50-layer residual network that utilizes skip connections to prevent the vanishing-gradient and degradation problems suffered by non-residual architectures at high depth; its organization takes the form of four stages of bottleneck blocks. Similar to VGG16, this model was fine-tuned from ImageNet weights, with a total of roughly 23.59 million parameters. Its structure is shown in **Figure 9**.

### 3.11 LSTM-CNN

**Figure 10:**
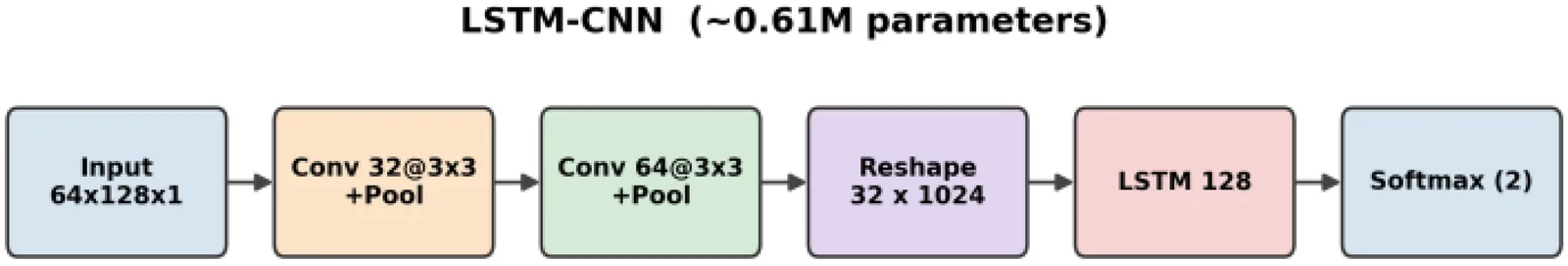
Structure of the LSTM-CNN architecture.

The LSTM-CNN is a model wherein there is implementation of a recurrent block, after the convolutional front end (Hochreiter and Schmidhuber, 1997). The network uses two convolutional layers (32 and 64 filters) with max pooling to extract localized spectral features. The feature map is reshaped into a sequence and then fed to a 128 unit LSTM, with a softmax layer making the prediction. The model’s capacity is only 0.61 million parameters. The design is depicted in **Figure 10**. We note explicitly that this network is included as a deliberately lightweight hybrid baseline rather than as a state-of-the-art sequence model; with only two convolutional layers and a 128-unit recurrent block it is far smaller than contemporary attention-based or transformer-based hybrids, and its position in the ranking should be interpreted accordingly.

### 3.12 Learning rate configuration

Among all the parameters that control the training of a deep network, the learning rate is usually the most important one, since it determines how big a step the optimiser takes each time it alters the weights (Goodfellow et al., 2016). If the learning rate is extremely high, then it can lead to overshooting and oscillations. Similarly, if the learning rate is extremely low, then the training will stall. However, it will show up in a decent spot, leaving the neural network underfit (Bengio, 2012). To train a network with SGD successfully it is vital to provide the right learning rate value. There is no one-size-fits-all learning rate to reach for. The accurate value varies according to architecture, depth, batch size, optimiser and contextual data itself (Bengio, 2012; Goodfellow et al., 2016). Optimisers that are adaptive like Adam were made to lessen this dependence making each parameter have their own step sizes based on running estimates of gradient first and second moments (Kingma and Ba, 2015). While these solutions are helpful, it does not mean the poorly chosen base rate is going away. This is because a poorly chosen base rate still scales every update and can push the model into instability or stagnation (Kingma and Ba, 2015).

A great deal of the existing literature addresses setting a time-varying learning rate during training. Smith suggested the use of cyclical learning rates which oscillate between the lower and upper bounds and a fast range test to identify these bounds (Smith, 2017). Loshchilov and Hutter introduced warm restarts with cosine annealing, periodically resetting the rate to assist the optimiser in avoiding sharp minima (Loshchilov and Hutter, 2017). Smith and Topin went further still, showing that a single training cycle with a very large maximum learning rate can speed up convergence dramatically, an effect they termed super-convergence (Smith, 2018). Other studies showed that raising the batch size can act much like lowering the learning rate (Smith et al., 2018). Two tricks that are commonly used in practice are stopping early if the validation score does not improve and reducing the learning rate when the validation loss flattens.

The vast majority of studies comparing the performance of automatic classifiers for heart sounds do not report how the learning rate was chosen, let alone how much their conclusions depend on it. When intentions are to compare architectures, it is a real weakness. This is the case as a network that appears worse may simply have been handed a bad rate. Rather, a network that appears better may simply have gotten lucky. To ensure a fair comparison, we trained each of the five networks at five learning rates covering two and a half orders of magnitude (1e-2, 1e-3, 5e-4, 1e-4 and 5e-5) with all else fixed. The Adam optimizer (Kingma and Ba, 2015) with categorical cross entropy loss was used for each run with two tricks: training stopped early when the validation loss stopped falling and halved rate when it plateaued. By taking the training rate as a pattern sweeping in the direction of deliberate aggression down to conservative, we may see both how it breaks and where each network works best. The largest rate produced constant destabilisation of several networks. The strongest results came from smaller values. This substantiates that the learning rate is one decisive factor for spectrogram-based heart-sound classification, as shown in Section 4.

**Table 4** summarises the complete training and experimental configuration common to all experiments.

**Table 4:** Training and experimental configuration.

Parameter	Value
Optimizer	Adam
Loss function	Categorical cross-entropy
Learning rates tested	1e-2, 1e-3, 5e-4, 1e-4, 5e-5
Maximum epochs	60
Batch size	32
Early stopping	patience 10 (monitor validation loss)
Learning-rate reduction	factor 0.5 on plateau (patience 5)
Class imbalance handling	Balanced class weights + SpecAugment (train only)
Input feature	Log-Mel spectrogram (64 x 128)
Framework	TensorFlow/Keras

### 3.13 Evaluation metrics

All the models were evaluated using 6 different performance measures which are based on the confusion matrix. Confusion Matrix counts true positives, true negatives, false positives, and false negatives. Here, we treat the abnormal class as positive. The proportion of recordings classified correctly is known as accuracy (Equation 1). Sensitivity (Equation 2) is the fraction of abnormal recordings caught by the model, whereas specificity (Equation 3) is the fraction of normal recordings caught by the model. The calculation of precision as reflected in Equation 4 shows how many of the flagged abnormal recordings are actually abnormal while the F1-score (see Equation 5) is the harmonic mean between precision and recall. The Matthews Correlation Coefficient (Equation 6) provides a balanced single score, which nevertheless remains meaningful when classes are skewed (the target numbers are imbalanced), and it range from minus one to plus one.

(1) $$Accuracy\;=\;\frac{TP\;+\;TN}{TP\;+\;TN\;+\;FP\;+\;FN}$$

(2) $$Sensitivity\;(Recall)\;=\;\frac{TP}{TP\;+\;FN}$$

(3) $$Specificity\;=\;\frac{TN}{TN\;+\;FP}$$

(4) $$Precision\;=\;\frac{TP}{TP\;+\;FP}$$

(5) $$F1-score\;=\;\frac{2\;\times \;TP}{2\;\times \;TP\;+\;FP\;+\;FN}$$

(6) $$MCC\;=\;\frac{TP\;\times \;TN\;-\;FP\;\times \;FN}{\sqrt{\left(TP+FP)(TP+FN)(TN+FP)(TN+FN\right)}}$$

Since accuracy on its own can flatter a model when the classes are unbalanced, we leaned on sensitivity, specificity, F1 and MCC instead. We also report the Area Under the ROC Curve (AUC): the ROC traces the true-positive rate against the false-positive rate across every threshold, and the AUC boils it down to one number, where 1.0 means perfect separation and 0.5 is no better than chance. Read together, these metrics give a fuller and more trustworthy picture of each model.

## 4 Results and discussion

Every model was trained with Adam and categorical cross-entropy, using early stopping and learning-rate reduction on plateau, for up to 60 epochs. With five networks and five learning rates this comes to 25 runs in total. We measured Accuracy, AUC, Sensitivity, Specificity, F1 and MCC on the held-out test set, where simply predicting the majority class would score 0.796. **Table 5** lists the full set of results.

**Table 5:** Evaluation indicators for all architectures across the five learning rates.

Model	LR	Acc	AUC	Sens	Spec	F1	MCC
LeNet5	0.01	0.204	0.500	1.000	0.000	0.339	0.000
LeNet5	0.001	0.893	0.936	0.657	0.954	0.714	0.653
LeNet5	0.0005	0.875	0.931	0.576	0.951	0.651	0.584
LeNet5	0.0001	0.893	0.931	0.687	0.946	0.723	0.659
LeNet5	5e-05	0.903	0.945	0.727	0.948	0.754	0.695
AlexNet	0.01	0.696	0.608	0.273	0.804	0.267	0.075
AlexNet	0.001	0.899	0.963	0.677	0.956	0.732	0.674
AlexNet	0.0005	0.914	0.961	0.697	0.969	0.767	0.720
AlexNet	0.0001	0.903	0.956	0.758	0.941	0.761	0.701
AlexNet	5e-05	0.901	0.947	0.657	0.964	0.730	0.677
VGG16	0.01	0.204	0.500	1.000	0.000	0.339	0.000
VGG16	0.001	0.920	0.964	0.808	0.948	0.804	0.754
VGG16	0.0005	0.922	0.963	0.727	0.972	0.791	0.748
VGG16	0.0001	0.922	0.970	0.778	0.959	0.802	0.754
VGG16	5e-05	0.924	0.970	0.758	0.966	0.802	0.757
ResNet50	0.01	0.899	0.941	0.727	0.943	0.746	0.684
ResNet50	0.001	0.897	0.955	0.798	0.922	0.760	0.696
ResNet50	0.0005	0.934	0.978	0.859	0.954	0.842	0.800
ResNet50	0.0001	0.879	0.913	0.616	0.946	0.674	0.604
ResNet50	5e-05	0.901	0.931	0.677	0.959	0.736	0.680
LSTM-CNN	0.01	0.825	0.853	0.505	0.907	0.540	0.435
LSTM-CNN	0.001	0.895	0.941	0.566	0.979	0.687	0.649
LSTM-CNN	0.0005	0.864	0.917	0.495	0.959	0.598	0.537
LSTM-CNN	0.0001	0.895	0.937	0.727	0.938	0.739	0.673
LSTM-CNN	5e-05	0.876	0.930	0.657	0.933	0.684	0.609

**Figure 11:**
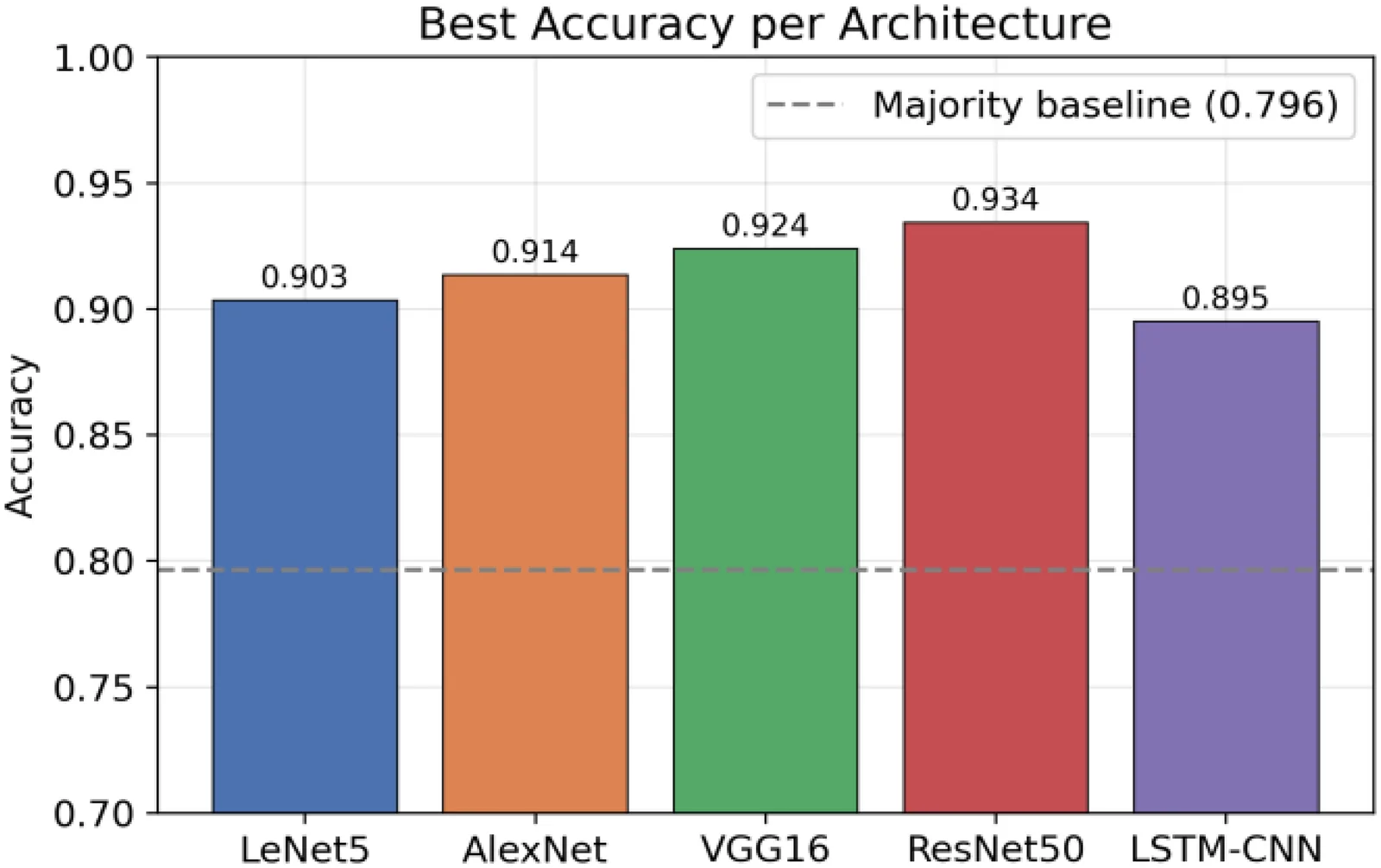
Best accuracy per architecture relative to the majority-class baseline.

**Figure 12:**
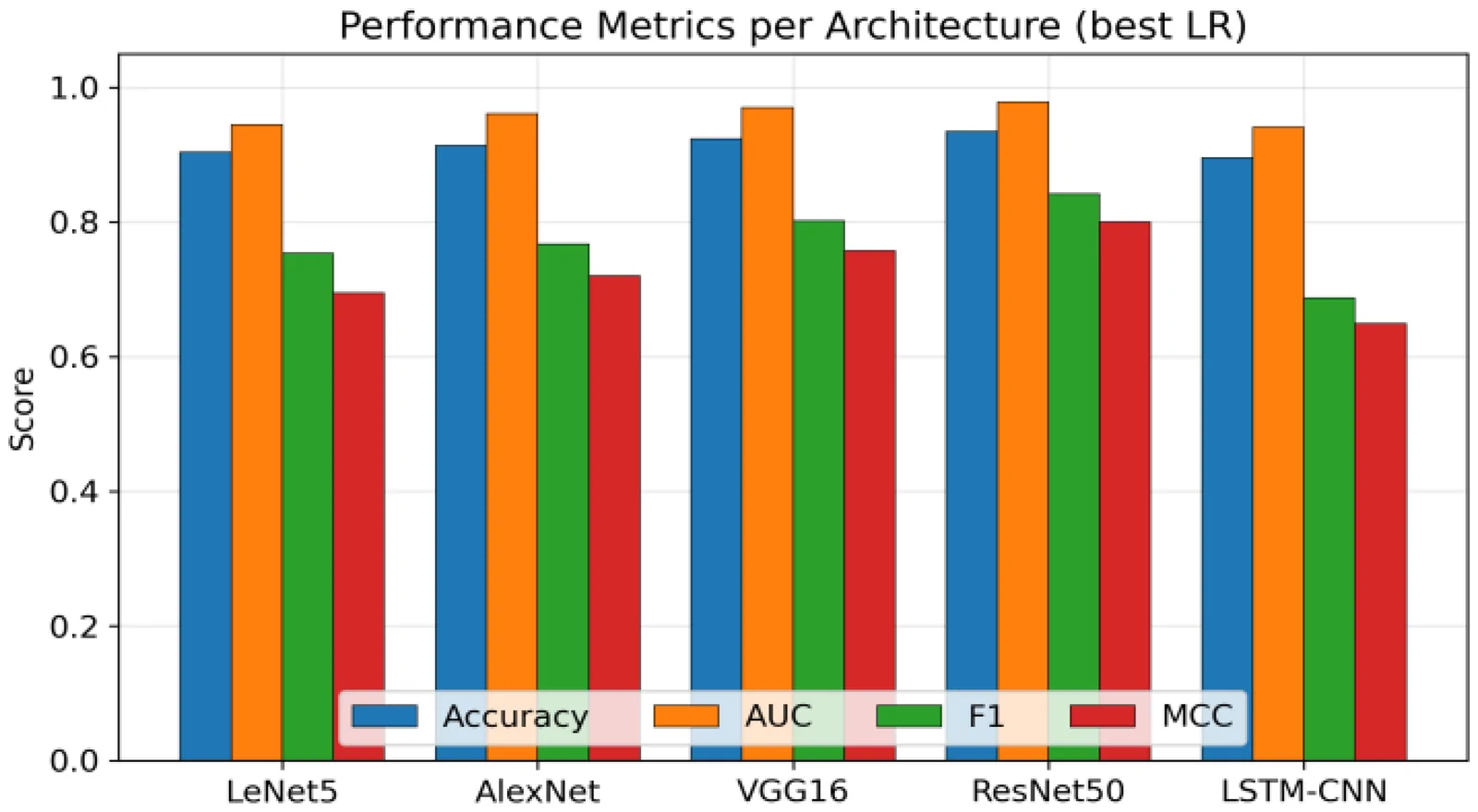
Accuracy, AUC, F1 and MCC for the best configuration of each architecture.

**Figure 13:**
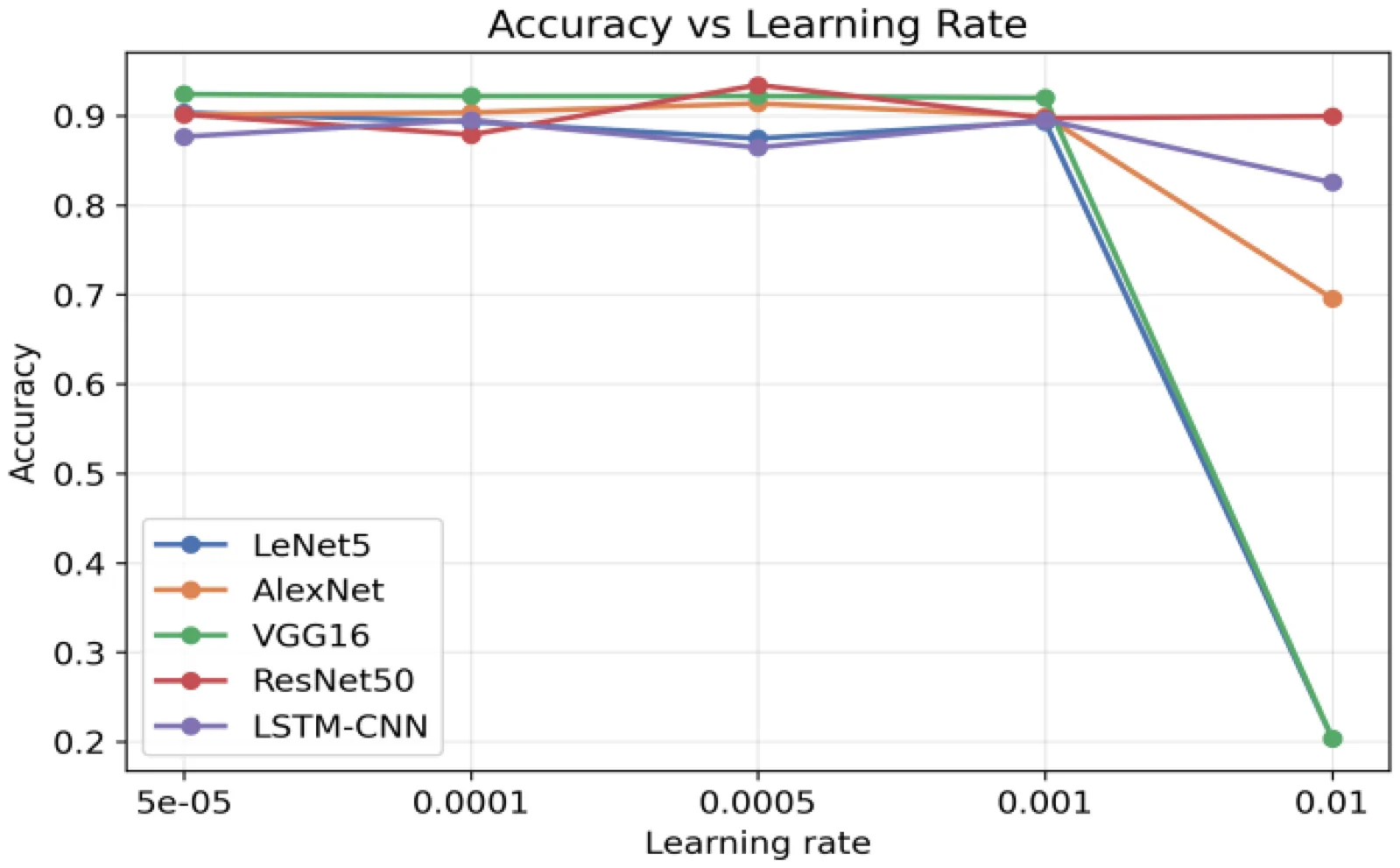
Effect of the learning rate on accuracy for each architecture.

**Figure 14:**
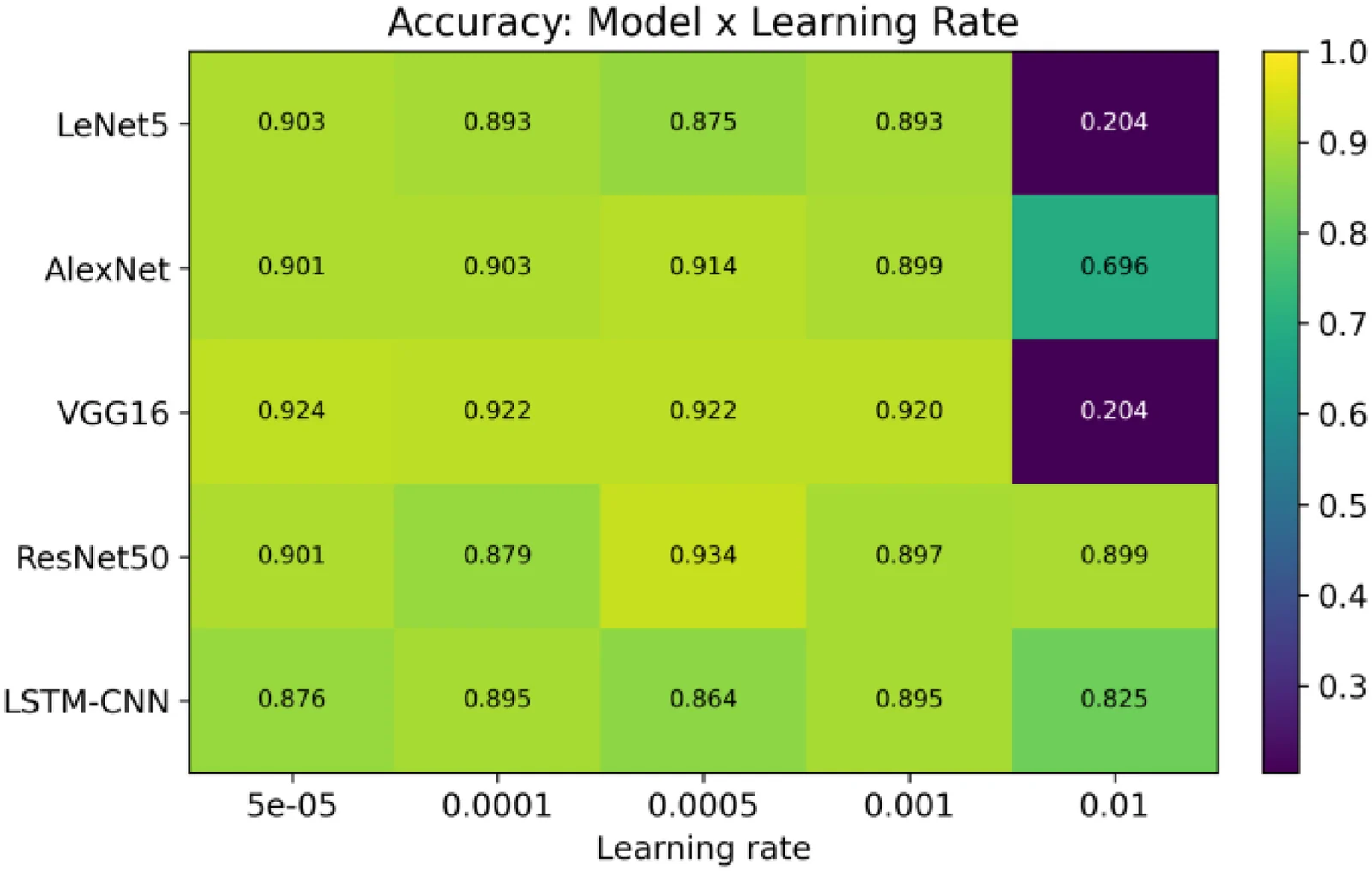
Accuracy heatmap across architectures and learning rates.

Two things stand out in **Table 5**. First, the highest learning rate (1e-2) is unstable: LeNet5 and VGG16 collapse to predicting a single class (Accuracy 0.204, AUC 0.500), and AlexNet is badly hurt too. Second, every network does best at a small rate, usually between 5e-4 and 5e-5. **Table 6** gathers the best configuration for each model, and **Figures 11**–**14** show the comparison visually.

**Table 6:** Best configuration per architecture (ranked by accuracy).

Model	Best LR	Acc	AUC	Sens	Spec	F1	MCC
ResNet50	5e-4	0.934	0.978	0.859	0.954	0.842	0.800
VGG16	5e-5	0.924	0.970	0.758	0.966	0.802	0.757
AlexNet	5e-4	0.914	0.961	0.697	0.969	0.767	0.720
LeNet5	5e-5	0.903	0.945	0.727	0.948	0.754	0.695
LSTM-CNN	1e-4	0.895	0.937	0.727	0.938	0.739	0.673

**Figure 15:**
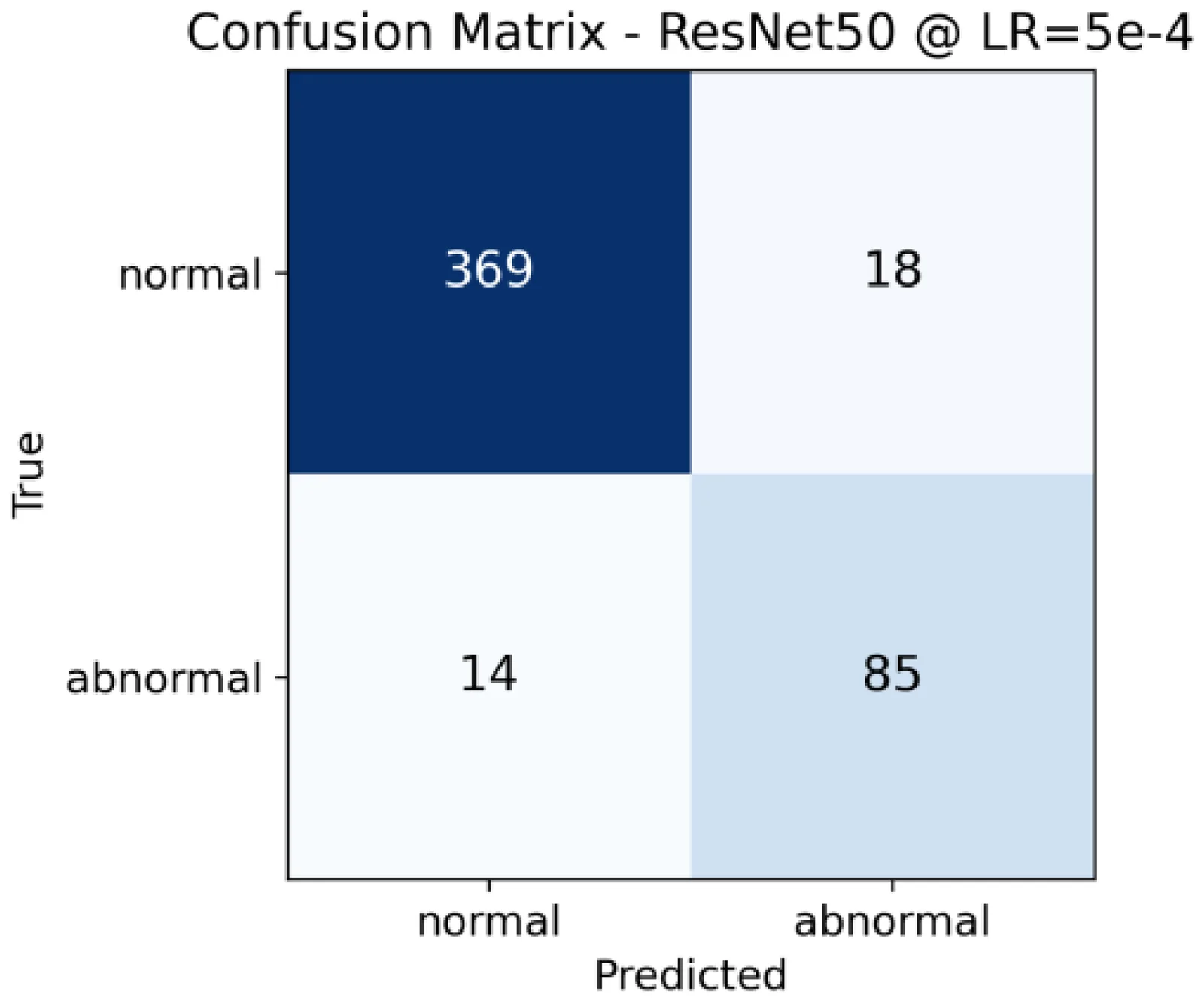
Confusion matrix of the best model (ResNet-50, LR = 5e-4).

**Figure 16:**
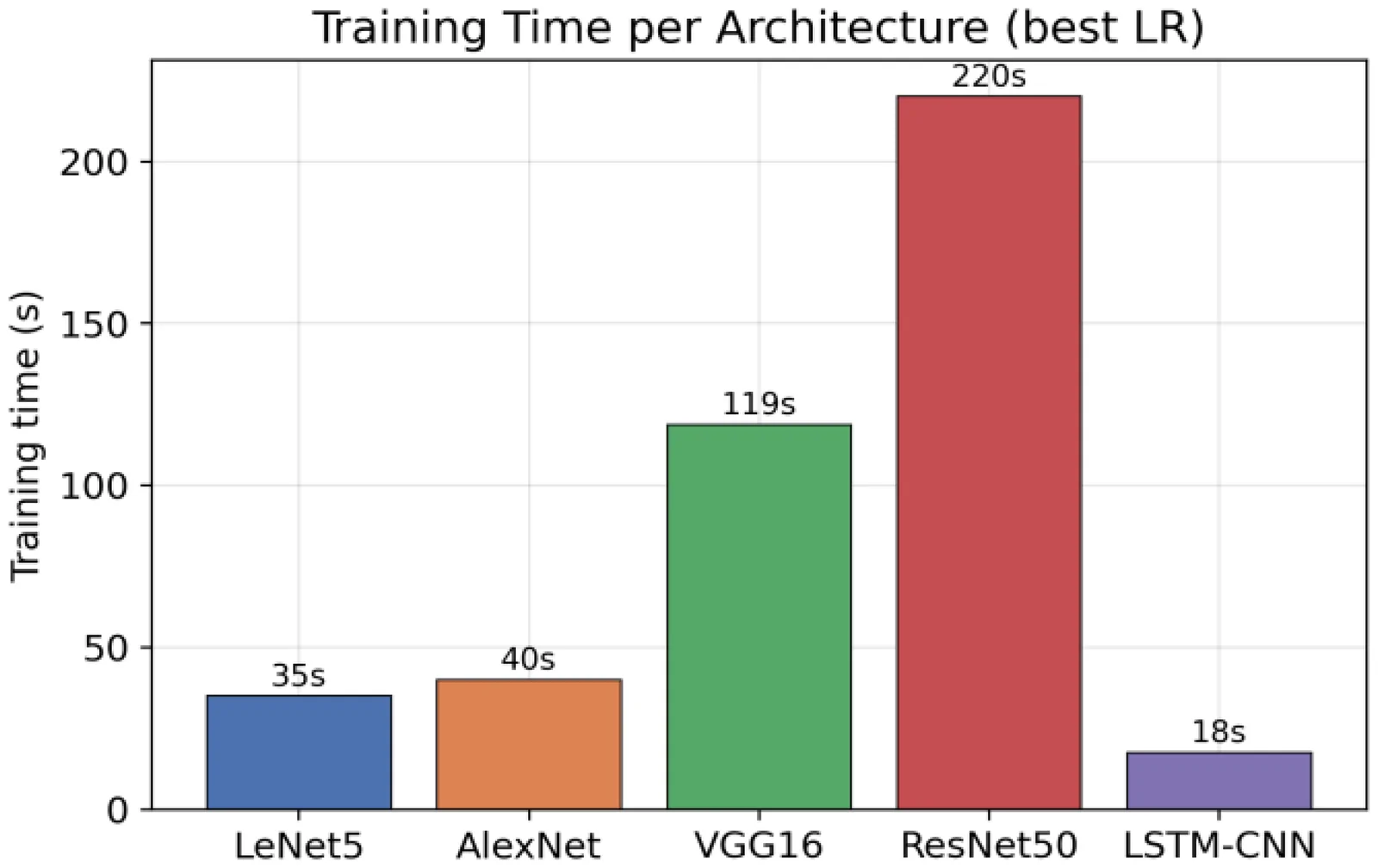
Training time per architecture (best configuration).

On this single hold-out split ResNet-50 obtained the highest score, reaching 93.42% accuracy, 0.978 AUC, 0.859 sensitivity, 0.954 specificity, 0.842 F1 and 0.800 MCC at a learning rate of 5e-4, with VGG16 close behind at 92.39% (AUC 0.970) and AlexNet at 91.36%. The compact LeNet5 held its own at 90.33% while training in a fraction of the time, and the LSTM-CNN came last of the five at 89.51%. As the cross-validation and significance testing in Sections 4.8 and 4.9 show, however, the gap between the two leading architectures lies within run-to-run variation, so this ordering should not be read as evidence that ResNet-50 is definitively superior to VGG16. **Figure 15** gives the confusion matrix of the best model on this split, and **Figure 16** shows the training-time trade-off: LeNet5 finished in about 35 seconds, against several minutes for the deeper networks.

**Figure 17:**
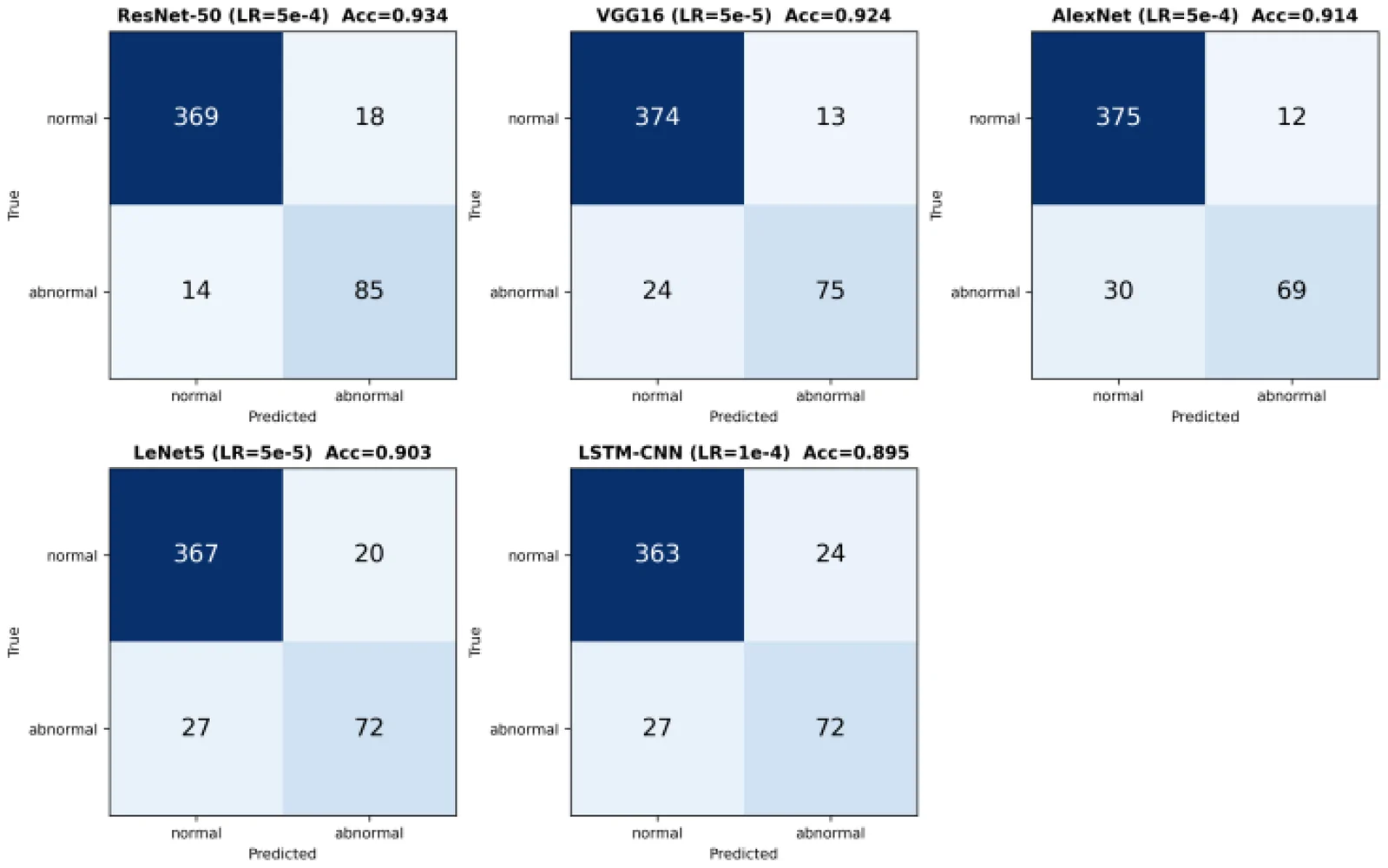
Confusion matrices of the best configuration of each architecture on the test set.

For completeness, **Figure 17** collects the confusion matrices of every model at its best setting. They tell a consistent story: the main error everywhere is calling an abnormal recording normal (the lower-left cell), which reflects how hard the minority abnormal class is to catch. On this split ResNet-50 achieves the most favourable balance, missing the fewest abnormal cases, although the difference with respect to VGG16 amounts to only a handful of recordings and is not statistically significant (Section 4.9).

Taken together, the results indicate that the deeper architectures, VGG16 and ResNet-50, generalise better on spectrogram-based heart sounds than the shallow or recurrent models, although the difference between these two is not statistically significant and their ordering changes between the single split and cross-validation. This is the opposite of what we found earlier for music-genre classification, where a shallower AlexNet led; the contrast suggests that the subtler and more variable patterns of heart sounds reward additional representational depth. It is worth emphasising that the benefit does not appear to come from transfer learning itself: the ablation in Section 4.10 shows that removing ImageNet initialisation changes performance by an amount comparable to random variation, whereas removing spectrogram augmentation costs four to five accuracy points. For this dataset the data-side design choice therefore matters more than the choice of pre-trained weights. The fact that every network collapses at the highest learning rate only underlines how much careful rate selection matters here.

**Table 7** sets our pipeline beside representative earlier work on PhysioNet 2016. With a cross-validated accuracy of 91.94% for VGG16 and 93.42% for ResNet-50 on the hold-out split, the leakage-free pipeline is competitive with published results while being obtained under a strict and reproducible protocol. Direct numerical comparison with the highest reported figures should nevertheless be made with caution. Several studies reporting near-perfect accuracy do not describe duplicate removal or patient-independent partitioning, and in our own experiments omitting those safeguards allowed several networks to reach a meaningless 100% accuracy on exactly this dataset. Recent attention-based and hybrid models, such as the convolutional block attention network of Huai et al. (2025) and the transformer-based framework of Al-Tam et al. (2024), report higher headline numbers than ours, but they are evaluated under different and generally unstated splitting protocols, so the comparison in **Table 7** should be read as indicative of the landscape rather than as a like-for-like ranking. Our contribution is accordingly less a new architecture than a demonstration of how much reported performance depends on evaluation discipline.

**Table 7:** Comparison with representative prior work on PhysioNet 2016.

Study	Dataset	Method	Acc	MCC
Potes et al. (2016)	PhysioNet 2016	Ensemble (AdaBoost + CNN)	0.860	—
Li et al. (2022)	PhysioNet 2016	Improved MFSC + ResNet	0.944	—
Huai et al. (2025)	PhysioNet 2016	CNN + CBAM	0.987	—
Deperlioglu (2021)	PhysioNet 2016	Stacked autoencoder	0.996	—
This work	PhysioNet 2016	ResNet-50 (log-Mel, TL)	0.934	0.800

### 4.1 Cross-validation analysis

**Figure 18:**
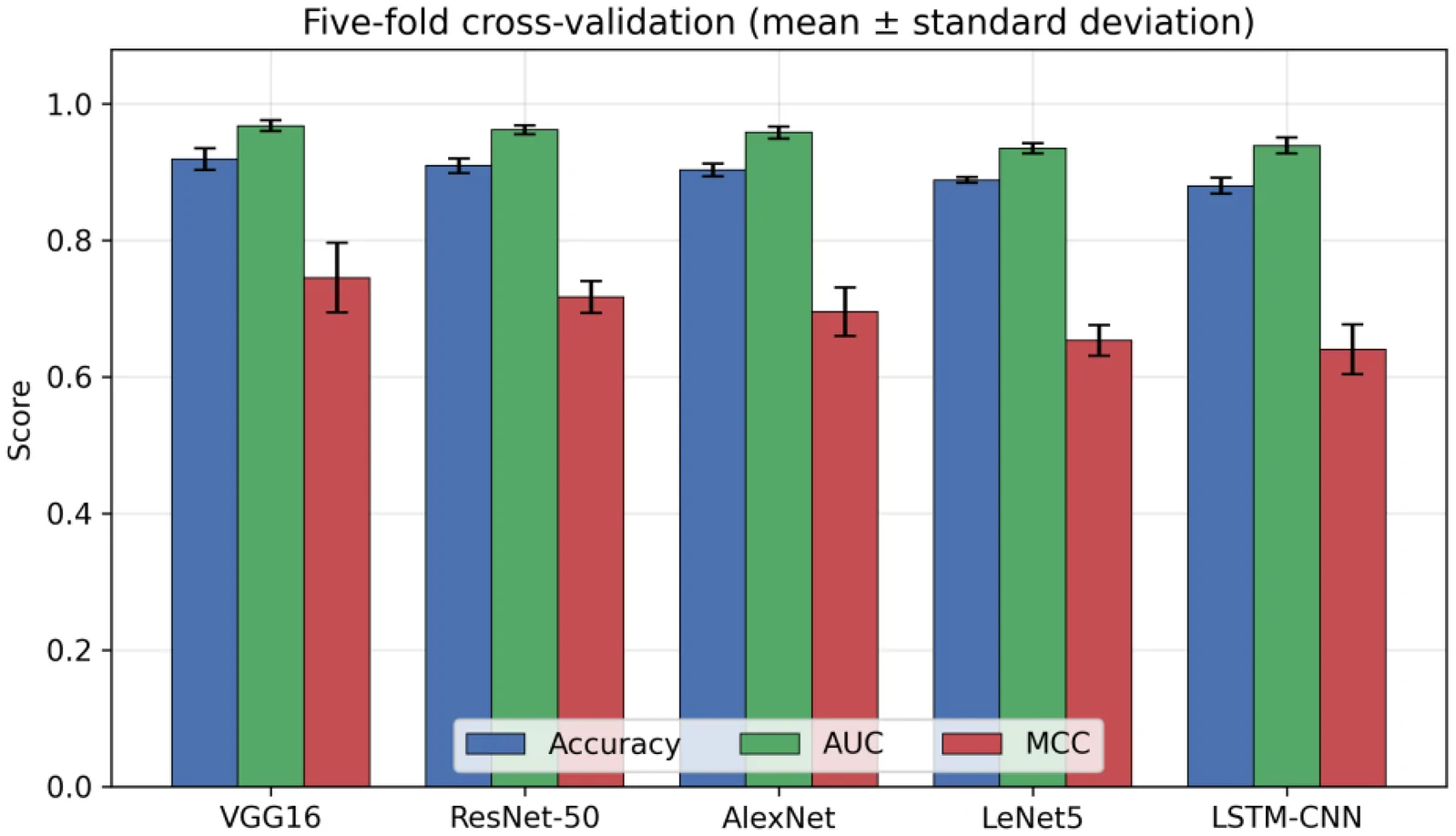
Five-fold cross-validation performance of the five architectures (mean ± standard deviation).

**Figure 19:**
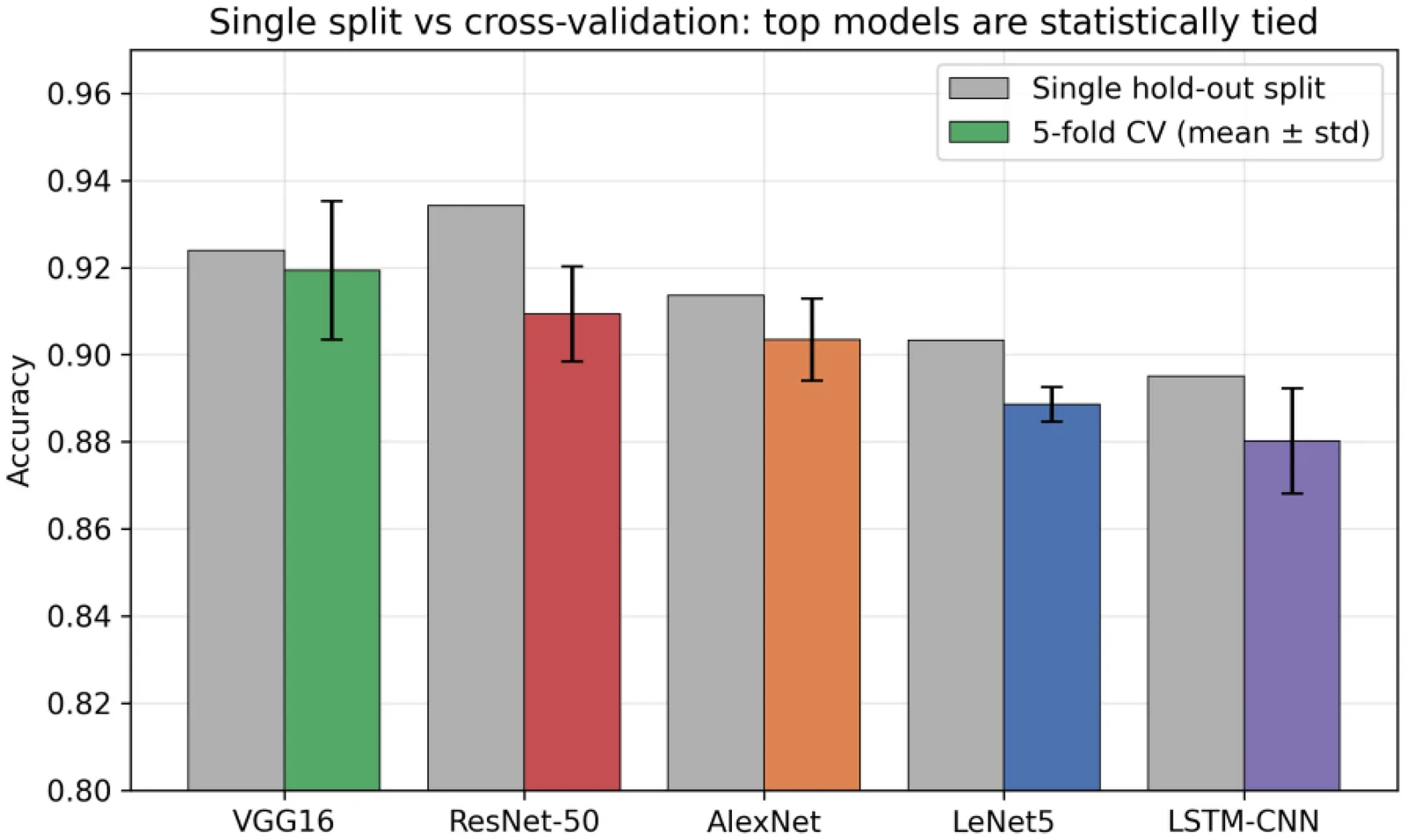
Single hold-out split compared with five-fold cross-validation. The leading architectures overlap within one standard deviation.

Because a single hold-out split can reward a fortunate random seed, we repeated the whole evaluation with stratified five-fold cross-validation. Each architecture was trained at its best learning rate on every fold, and the mean and standard deviation of each metric are reported in **Table 8** and illustrated in **Figure 18**. Two conclusions follow. First, the results are stable: the standard deviation of accuracy never exceeds 0.016, so the ranking is not an artefact of seed variation. Second, the ordering of the two strongest models changes. Under cross-validation VGG16 attains the highest mean accuracy (0.9194 ± 0.0159, AUC 0.9677, MCC 0.7454), slightly ahead of ResNet-50 (0.9093 ± 0.0109), whereas the single split had favoured ResNet-50. **Figure 19** places the two protocols side by side and shows that the difference between the leading architectures lies within one standard deviation. We therefore no longer claim a single best architecture, and instead report VGG16 and ResNet-50 as jointly the strongest models, with AlexNet, LeNet5 and the LSTM-CNN following in that order.

**Table 8:** Five-fold cross-validation results (mean ± standard deviation).

Model	Best LR	Accuracy	AUC	Sensitivity	Specificity	F1	MCC
VGG16	5e-5	0.9194 ± 0.0159	0.9677 ± 0.0080	0.7293 ± 0.0964	0.9685 ± 0.0222	0.7859 ± 0.0525	0.7454 ± 0.0510
ResNet-50	5e-4	0.9093 ± 0.0109	0.9623 ± 0.0066	0.7293 ± 0.0681	0.9557 ± 0.0262	0.7672 ± 0.0213	0.7166 ± 0.0234
AlexNet	5e-4	0.9034 ± 0.0094	0.9579 ± 0.0085	0.7233 ± 0.0564	0.9499 ± 0.0081	0.7537 ± 0.0323	0.6956 ± 0.0352
LeNet5	5e-5	0.8886 ± 0.0040	0.9348 ± 0.0072	0.7068 ± 0.0545	0.9355 ± 0.0093	0.7217 ± 0.0231	0.6534 ± 0.0228
LSTM-CNN	1e-4	0.8802 ± 0.0121	0.9390 ± 0.0114	0.7308 ± 0.0645	0.9188 ± 0.0209	0.7142 ± 0.0298	0.6405 ± 0.0364

### 4.2 Statistical significance testing

To establish whether the small differences between the leading architectures are meaningful, McNemar’s exact test was applied to the paired predictions of the models on the common held-out test set. The results are given in **Table 9**. ResNet-50 and VGG16 disagree on only 24 recordings (9 correct for ResNet-50 only, 15 for VGG16 only), giving p = 0.3075; the comparison between ResNet-50 and AlexNet gives p = 0.1214. Neither difference reaches the conventional 0.05 threshold. Taken together with the cross-validation, this demonstrates that the roughly one-point accuracy gap separating the top architectures is not statistically significant and should not be interpreted as evidence that one network is superior.

**Table 9:** McNemar’s exact test between the leading architectures on the held-out test set.

Comparison	Correct for A only	Correct for B only	p-value	Significant at 0.05
ResNet-50 (A) vs VGG16 (B)	9	15	0.3075	No
ResNet-50 (A) vs AlexNet (B)	22	12	0.1214	No

### 4.3 Ablation study

**Figure 20:**
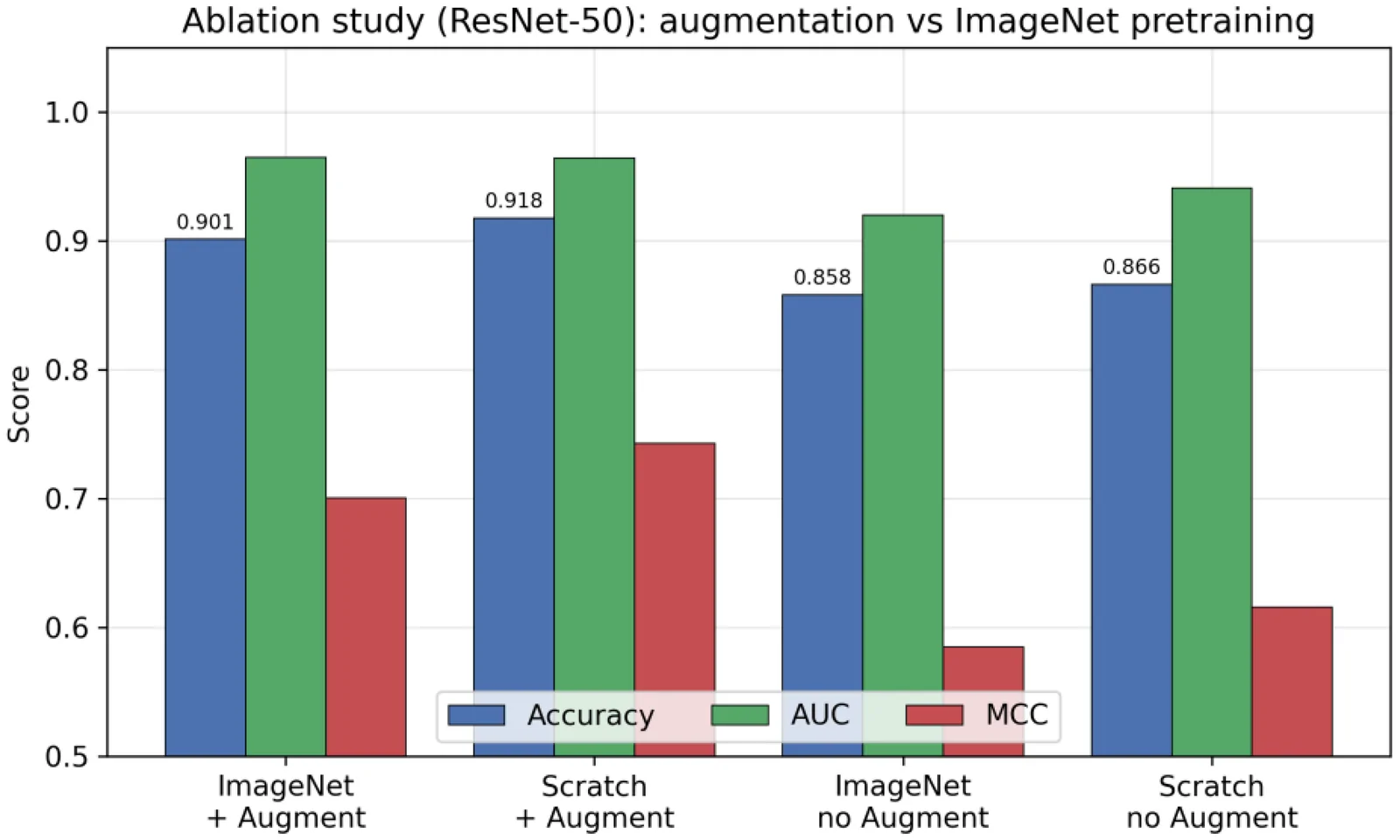
Ablation study on ResNet-50. Augmentation dominates; ImageNet pre-training shows no consistent benefit.

An ablation study was carried out on ResNet-50 to separate the contribution of the two main design choices in the pipeline, namely ImageNet pre-training and spectrogram augmentation. The four configurations are compared in **Table 10** and **Figure 20**. Augmentation is clearly the decisive factor: removing it costs between four and five accuracy points and reduces the MCC by 0.12 to 0.13 in both pre-training settings. The effect of ImageNet pre-training, by contrast, is small and inconsistent; the network trained from scratch actually scored marginally higher (0.9177 against 0.9012). Since this gap is of the same order as the run-to-run variation observed in Section 4.8, we conclude only that transfer learning provided no consistent benefit on this dataset, and we do not claim that random initialisation is superior. This tempers the assumption, common in the literature, that ImageNet features are always helpful for spectrogram-based biomedical audio.

**Table 10:** Ablation study on ResNet-50 (held-out test set).

Configuration	Accuracy	AUC	Sensitivity	Specificity	F1	MCC
ImageNet + augmentation	0.9012	0.9646	0.7778	0.9328	0.7624	0.7003
From scratch + augmentation	0.9177	0.9640	0.7778	0.9535	0.7938	0.7427
ImageNet, no augmentation	0.8580	0.9201	0.7172	0.8941	0.6730	0.5846
From scratch, no augmentation	0.8663	0.9409	0.7576	0.8941	0.6977	0.6157

### 4.4 External validation on CirCor DigiScope 2022

**Figure 21:**
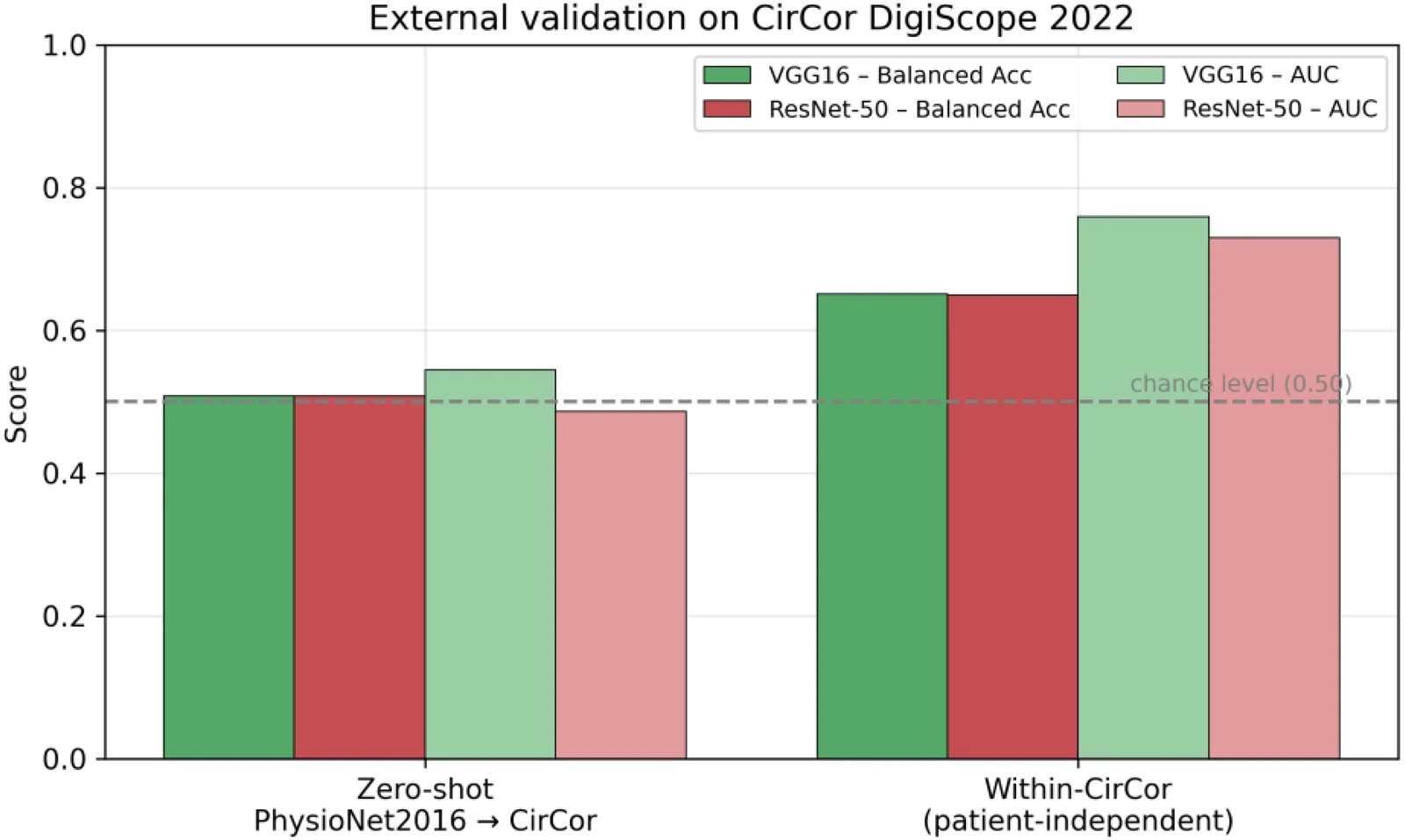
External validation on CirCor DigiScope 2022. Zero-shot transfer is at chance level; the retrained pipeline performs above baseline.

All results presented so far come from a single corpus, so we additionally evaluated the two leading architectures on the CirCor DigiScope 2022 database (Oliveira et al., 2022), a newer and considerably more challenging paediatric collection recorded during mass screening campaigns. Two experiments were run and are summarised in **Table 11** and **Figure 21**. In the first, models trained on PhysioNet 2016 were applied to CirCor without any adaptation. Transfer failed almost completely: balanced accuracy was 0.508 for both networks and the MCC was close to zero, which is indistinguishable from chance. Inspection of the predictions shows the models label more than ninety per cent of the recordings as abnormal, so plain accuracy (0.25) falls far below the majority-class baseline of 0.795 and is not a meaningful summary here. Two factors contribute. The domain differs sharply, since PhysioNet 2016 is predominantly adult and clinical whereas CirCor is paediatric and acquired in the field; and, more fundamentally, the label definitions differ, because PhysioNet 2016 marks a confirmed clinical diagnosis while the CirCor annotation available to us records the presence of an audible murmur. The two targets are related but not equivalent, and this result should therefore be read as evidence of combined domain and label shift rather than as a failure of the architectures themselves.

**Table 11:** External validation on CirCor DigiScope 2022. Balanced accuracy is reported because plain accuracy is misleading when a model collapses onto one class.

Model	Train	Test	n	Baseline	Accuracy	Balanced Acc.	AUC	Sensitivity	Specificity	MCC
VGG16	PhysioNet 2016	CirCor 2022	3007	0.7951	0.2511	0.5080	0.5445	0.9432	0.0728	0.0253
ResNet-50	PhysioNet 2016	CirCor 2022	3007	0.7951	0.2627	0.5081	0.4864	0.9237	0.0924	0.0228
VGG16	CirCor 2022	CirCor 2022	603	0.8109	0.8541	0.6510	0.7593	0.3246	0.9775	0.4370
ResNet-50	CirCor 2022	CirCor 2022	603	0.8109	0.8507	0.6490	0.7301	0.3246	0.9734	0.4231

In the second experiment the complete pipeline, including content-hash de-duplication and a strictly patient-independent split, was retrained and tested within CirCor. Here performance is modest but clearly above chance: VGG16 reaches 0.8541 accuracy, 0.7593 AUC and 0.4370 MCC against a majority baseline of 0.8109, and again edges ahead of ResNet-50. Sensitivity remains low at 0.3246, meaning roughly two thirds of murmurs are missed, which we regard as the main clinical limitation of the present models on this harder corpus. The comparison between the two datasets is itself informative: the same pipeline that reaches an AUC of about 0.97 on PhysioNet 2016 achieves only about 0.76 on CirCor, which supports the view that results obtained on a single benchmark can substantially overstate real-world performance.

### 4.5 Computational complexity and deployment considerations

Beyond accuracy, the practical value of a screening tool depends on how expensive it is to train and to run. **Table 3** already lists the parameter counts, which span almost two orders of magnitude, from 0.61 million for the LSTM-CNN and 0.82 million for LeNet5 up to 23.59 million for ResNet-50. Training cost follows the same pattern: at their best settings LeNet5 converged in roughly 35 seconds and the LSTM-CNN in about 34 seconds, whereas VGG16 and ResNet-50 required approximately 119 and 220 seconds respectively on a single GPU, a factor of three to six more. The accuracy purchased with that extra computation is comparatively small: LeNet5 reaches 0.8886 mean accuracy under cross-validation against 0.9194 for VGG16, a gap of about three points for roughly eighteen times the parameters. For deployment on a digital stethoscope, a smartphone or a low-power edge board, this trade-off matters. Converting the compact models to an eight-bit quantised TensorFlow Lite representation yields a file of a few hundred kilobytes that can perform inference in real time on commodity hardware without a GPU, whereas ResNet-50 would require either a more capable device or a server-side deployment with the attendant connectivity and privacy implications. A practical system might therefore favour a compact network at the point of care, reserving the larger architectures for centralised review, and we regard a systematic latency and energy study on real devices as a natural extension of this work.

### 4.6 Applicability of the leakage-free protocol to other biomedical datasets

The evaluation protocol proposed here is deliberately independent of the modality. It rests on two ideas: exact duplicates are identified by hashing the decoded signal rather than by trusting file names or the folder structure supplied with a corpus, and the split is then made at the level of the patient rather than the individual recording. Neither step assumes anything specific about heart sounds, and both address failure modes that recur across biomedical collections. Public electrocardiogram, electroencephalogram and lung-sound archives are frequently assembled from several sources and often redistribute the same signal in more than one partition, while imaging datasets routinely contain multiple slices, views or follow-up studies belonging to one individual. In every one of these situations a naive record-level split allows a model to encounter the same underlying subject during training and testing, which inflates the reported score without any genuine gain in generalisation. Our own experiments illustrate how large that inflation can be, since omitting the de-duplication step allowed several architectures to reach an implausible one hundred per cent accuracy on this dataset. The protocol also transferred without modification to CirCor DigiScope in Section 4.11, where grouping by patient is essential because each participant contributes several auscultation positions. We therefore suggest that content-hash de-duplication followed by subject-level partitioning be adopted as a routine and inexpensive safeguard whenever benchmark performance is reported on public biomedical data.

### 4.7 Limitations

Several limitations should be acknowledged. The sensitivity of every model remains lower than its specificity, so abnormal recordings are still missed more often than normal ones; on CirCor in particular the sensitivity of 0.3246 would be inadequate for unsupervised clinical screening, and threshold calibration or cost-sensitive training deserves further study. The LSTM-CNN was included as a deliberately lightweight hybrid baseline of two convolutional layers followed by a 128-unit recurrent block, and it is therefore not representative of current attention-based or transformer-based sequence models; its position at the bottom of the ranking should be read with that in mind. The cross-dataset experiment is further limited by the fact that the CirCor annotation available to us encodes murmur presence rather than a confirmed clinical outcome, so domain shift and label shift cannot be fully separated. Finally, the ablation was run once per configuration, and because run-to-run variation is of a similar magnitude to the pre-training effect, that particular comparison should be treated as indicative rather than conclusive.

## 5 Conclusion and future works

In this study we compared five CNN architectures for classifying a heart sound as normal or abnormal. The experiments used the PhysioNet/CinC 2016 database and, specifically, log-Mel spectrograms extracted from the PCG signals. The architectures examined were LeNet5, AlexNet, VGG16, ResNet-50 and a hybrid LSTM-CNN. Five learning rates were run per network and judged by six metrics, all under a leakage-free protocol that removes duplicates by content hashing and keeps patients apart across the splits. Under five-fold cross-validation VGG16 achieved the highest mean accuracy (91.94%, AUC 0.968, MCC 0.745), marginally ahead of ResNet-50, and McNemar’s test showed that the difference between the leading architectures is not statistically significant; we therefore report the two deeper networks as jointly strongest rather than naming a single winner. An ablation study identified spectrogram augmentation, not ImageNet pre-training, as the decisive design choice, and external validation on CirCor DigiScope 2022 showed that zero-shot transfer to a newer paediatric corpus performs at chance level while the retrained pipeline exceeds the majority baseline. We also observed how strongly the learning rate affects stability, with the highest value collapsing several models, and how omitting duplicate removal can manufacture misleadingly perfect scores. Several directions remain open. Explainable-AI techniques such as Grad-CAM and SHAP would allow the spectro-temporal regions driving each decision to be visualised and audited by clinicians, which we regard as a prerequisite for clinical adoption; attention mechanisms and richer multi-representation inputs combining STFT, Mel and wavelet features, segmentation of individual heartbeats before classification, domain-adaptation methods to close the cross-dataset gap identified in Section 4.11, and porting the compact models to edge devices for real-time tele-health screening are further natural extensions of this work.

## Data Availability

The original contributions presented in the study are included in the article/supplementary material. Further inquiries can be directed to the corresponding author.
